# Generalized and social anxiety disorder interactomes show distinctive overlaps with striosome and matrix interactomes

**DOI:** 10.1038/s41598-021-97418-w

**Published:** 2021-09-15

**Authors:** Kalyani B. Karunakaran, Satoko Amemori, N. Balakrishnan, Madhavi K. Ganapathiraju, Ken-ichi Amemori

**Affiliations:** 1 Supercomputer Education and Research Centre, Indian Institute of Science, Bangalore, India; 2 Institute for the Advanced Study of Human Biology, Kyoto University, Kyoto, Japan; 3 Department of Biomedical Informatics, School of Medicine, University of Pittsburgh, Pittsburgh, USA; 4 Intelligent Systems Program, School of Computing and Information, University of Pittsburgh, Pittsburgh, USA

**Keywords:** Neuroscience, Diseases of the nervous system, Molecular neuroscience, Stress and resilience, Genetic association study, Neurodevelopmental disorders, Proteome informatics, Systems biology, Anxiety, Obsessive compulsive disorder, Post-traumatic stress disorder, Computational biology and bioinformatics, Genome informatics

## Abstract

Mechanisms underlying anxiety disorders remain elusive despite the discovery of several associated genes. We constructed the protein–protein interaction networks (interactomes) of six anxiety disorders and noted enrichment for striatal expression among common genes in the interactomes. Five of these interactomes shared distinctive overlaps with the interactomes of genes that were differentially expressed in two striatal compartments (striosomes and matrix). Generalized anxiety disorder and social anxiety disorder interactomes showed exclusive and statistically significant overlaps with the striosome and matrix interactomes, respectively. Systematic gene expression analysis with the anxiety disorder interactomes constrained to contain only those genes that were shared with striatal compartment interactomes revealed a bifurcation among the disorders, which was influenced by the anterior cingulate cortex, nucleus accumbens, amygdala and hippocampus, and the dopaminergic signaling pathway. Our results indicate that the functionally distinct striatal pathways constituted by the striosome and the matrix may influence the etiological differentiation of various anxiety disorders.

## Introduction

Anxiety is a mental state evoked in anticipation of a potential threat. Individuals may either exhibit acute levels of anxiety in response to an immediate threat or persistent levels of heightened anxiety (trait anxiety) in non-threatening situations as part of a ‘neurotic’ personality trait^1^. While both these non-pathological forms of anxiety may have evolved to protect the individual from potential dangers, the latter predisposes the individual to a range of anxiety disorders (ADs), depression, or both^2^. ADs, including generalized anxiety disorder (GAD), social anxiety disorder (SAD), specific phobia, post-traumatic stress disorder (PTSD) and obsessive–compulsive disorder (OCD), affect 284 million people (63% females, 2.5–7% variation by country), and are among the most prevalent mental health and neurodevelopmental disorders (WHO and IHME, 2017)^3^. About 31% of U.S. adults experience at least one AD during their lifetime^1,4^. ADs exhibit substantial familial aggregation with 30–50% heritability^5,6^, and about 50% comorbidity of various AD types^7^. Despite the discovery of several genes associated with these disorders through linkage^8,9^ and genome-wide association studies^10–14^, the neurobiological implications of their genetic architectures remain elusive.

Several factors may reflect the distinct etiologies of various ADs, including different diagnostic definitions, regionally-specific neural activity and region-specific gene expression in the brain. By definition, SAD symptoms are conditional and ‘externally’ provoked by exposure to social situations^15^. In contrast, GAD is ‘internally’ provoked in the absence of any apparent anxiety-inducing event^15^. Regionally-specific neural activity has been associated with these disorders. For example, pregenual anterior cingulate cortex (ACC) of GAD patients showed hyperactivity correlated with their treatment responses^16^, whereas bilateral amygdala showed hyperactivity in response to emotional stimuli in SAD patients^17^. Neural activity patterns within specific brain regions and among anatomically/functionally connected regions may underlie cognitive and emotional states in anxiety and can be correlated with transcriptional profiles^18–24^. It is thus possible that psychiatric morbidities such as ADs that are strongly driven by specific brain regions or networks would exhibit abnormalities in region-specific transcriptional signatures. Although anxiety-linked regional gene expression has been examined in post-mortem human brain tissues, blood samples and pharmacogenomic animal models^25–29^, the functional consequences of such regional specificities remain unclear. In the current study, we examined ADs within the mechanistic framework of the protein–protein interaction (PPI) network (or the ‘interactome’), which has revealed higher-order relationships in the genetic structures of complex disorders^30–33^.

Protein–protein interactions (PPIs) drive the cellular machinery by facilitating a variety of biological processes, including signal transduction, the formation of cellular structures and enzymatic complexes. The effect of genetic mutations and abnormal gene expression may affect proteins and PPIs, posing deeper implications for disease development, such as multiple pathophenotypes that cannot be attributed to a single genotype in a disease^34^. Such effects can be explained through the analysis of the interactome, which allows examination of shared genetics, biological pathways and symptomatology^30–33^.

**Figure 1:**
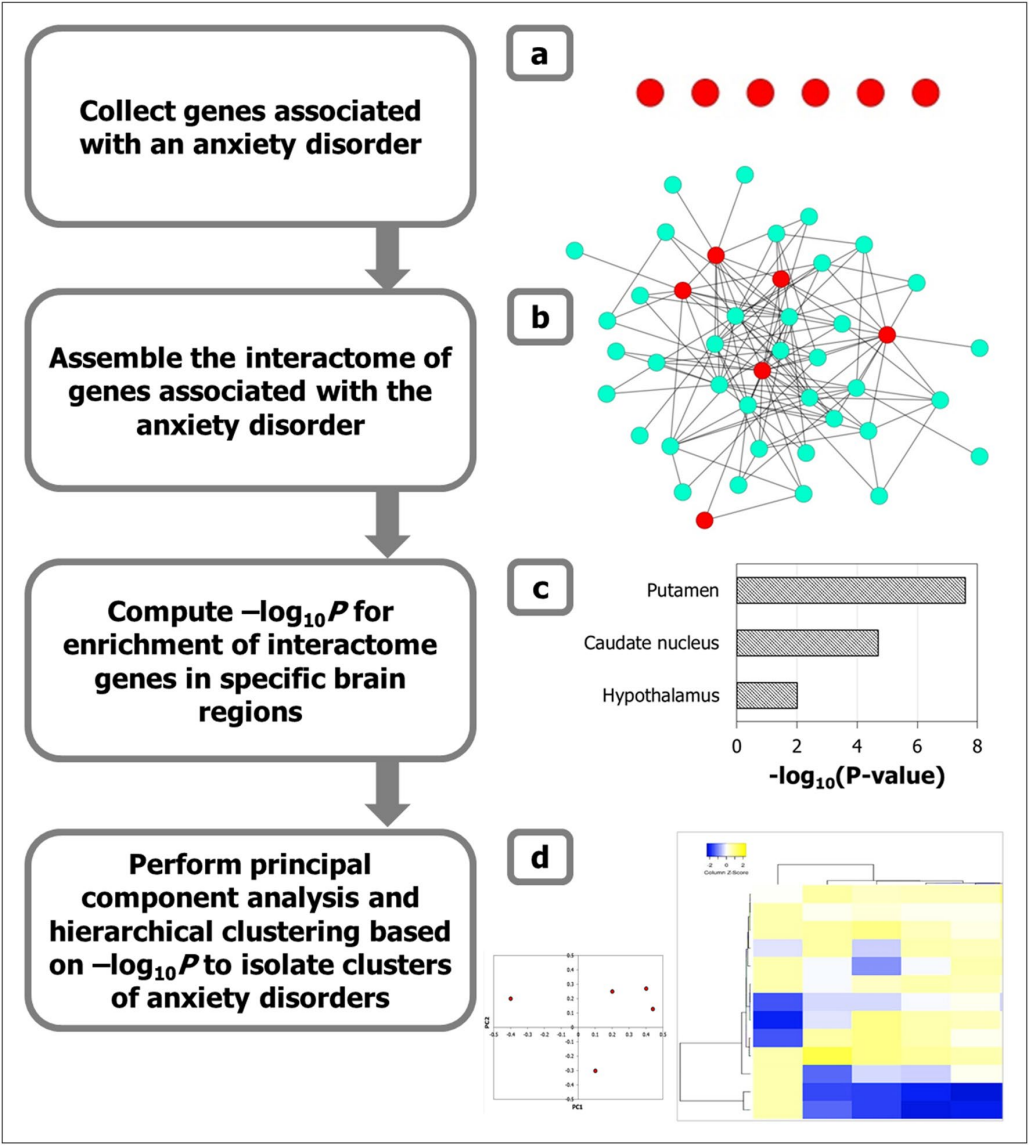
Methodology to identify regional specificity of ADs in the brain. (**a**) Genes associated with six types of ADs were compiled (depicted as red nodes), and (**b**) their protein interactomes assembled by curating the interactions of the proteins encoded by them with other proteins (i.e. interactors depicted as cyan colored nodes in the network diagram). These protein–protein interactions or PPIs are depicted as edges in the network. (**c**) The enrichment of the individual ADIs with genes showing medium/high expression (TPM ≥ 9) in specific brain regions were computed, and the statistical significance of these enrichments were calculated as negative logarithm of *p* values (i.e. –log_10_P). TPM = transcripts per million. (**d**) Principal component analysis (PCA) was performed to identify specific grouping patterns from the data matrix of –log_10_P of enrichment of each ADI in specific brain regions. Principal components which explain a large percentage of the variance observed across this data matrix were identified, and the component loadings denoting the correlation of the original variables (− log_10_P of specific brain regions) with the principal components were examined to interpret the observed patterns. The data matrix was also subjected to hierarchical clustering to delineate closely related groups of ADs. In the heat map, regions were colored according to the z-scores indicating their mean enrichment in the ADI. The z-scores indicate the number of standard deviations that separate a given *p* value from the mean. High z-scores correspond to high enrichment for the specific region.

We constructed the interactomes for six types of ADs (GAD, SAD, OCD, specific phobia, panic disorder and PTSD interactomes) using genes associated with each AD in biomedical literature as starting points. We identified the transcriptional profiles that characterized each of the anxiety disorder interactomes (ADIs) by computing the enrichment of the genes contained in these ADIs for high/moderate expression in specific human brain regions (Fig. 1a-c). For example, enrichment of a specific ADI for ACC-expressed genes was determined by comparing the distribution of ACC-expressed genes in that ADI against the background distribution of ACC-expressed genes among all the genes expressed in the brain. We then performed principal component and hierarchical clustering analyses to characterize region-wise expression patterns of ADIs and delineate AD groups (Fig. 1d).

We observed that the genes that commonly co-occur in all the ADIs were strikingly enriched for expression in the striatum. We conducted a detailed interactome-based analysis of two striatal subdivisions called striosomes and matrix. The striatum is the primary input of the basal ganglia and is critical to motor control and motivated behaviors^35^. The striatum itself is histologically and neurochemically segregated into the striosome (or patch) and matrix compartments, which have differential gene expression signatures, anatomical connections and developmental patterns^36^. Striosomes are labyrinthine structures found embedded within the extra-striosomal matrix^37^. Striosomes express elevated levels of the µ-opioid receptors (MORs) in rodents^38^ and Kv4 potassium channel subunit (KChIP1) in primates^37^, whereas matrix expresses elevated levels of calbindin^39^, somatostatin^40^, encephalin^41^ and acetylcholinesterase^42^. Striosomes are preferentially innervated by cortical areas implicated in limbic and evaluative processes such as the caudal orbitofrontal cortex (cOFC), pregenual anterior cingulate cortex (pACC)^43–45^ in primates, and prelimbic cortex (PL) in rodents^46^. The medium spiny neurons (MSNs) in the striosome and matrix send projections to the substantia nigra pars reticulata and the external and internal segments of the globus pallidus, but only striosome MSNs have projections to the dopamine neurons in substantia nigra pars compacta^47,48^.

It is noteworthy that both the striatal subdivisions are preferentially innervated by regions that may govern various aspects of anxiety. Both striosomes and the matrix arise from progenitor cell populations constituting the lateral ganglionic eminence^47^. However, striosomal neurons migrate out into the striatum from the lateral ganglionic eminence earlier than the matrix^47^. The functions of striosomes and matrix are yet to be fully elucidated. Nevertheless, studies have implicated them in reward-guided decision making and motivational conflict during cost–benefit decision making^44,46,49–51^, and demonstrated their differential involvement in Huntington’s disease^52^, Parkinson’s disease^53^, motor stereotype^54^, and drug addiction^47^. Interestingly, the striosome/matrix interactomes (SMIs), assembled using genes differentially expressed in the striosome and the matrix, showed preferential overlap with specific ADIs. Further, the genes shared between specific ADIs and SMIs showed discrete expression patterns, which allowed us to cluster the various ADs. Our findings implicate striatum as one of the focal points of etiological differentiation of ADs by showing that region-specific expression patterns underlying these disorders emerge only when the ADIs are constrained to include those genes that are shared with the SMIs.

## Results

### Expression of ADI genes in brain regions

Genes associated with six types of ADs (from ref^55^ Fig. 1. Suggested scheme for exploring a suspected anxiety disorder), namely, PTSD, OCD, GAD, SAD, specific phobia and panic disorder, were extracted from DisGeNET^56^ (Supplementary Table S1). Note that (a) DisGeNET catalogs gene-disease associations described in animal models such as rats and mice, in addition to those described in human studies, and (b) many of the genes cataloged in DisGeNET may not share a causal relationship with the disease, and may instead only be associated with disease susceptibility or endophenotypes. Using RNA-sequencing data of 13 postnatal human brain regions obtained from GTEx^57^, we attempted to identify whether these genes were enriched for expression in a specific brain region in a statistically significant manner. Genes with high/medium expression (transcripts per million (TPM) ≥ 9) in these 13 brain regions that were not housekeeping genes (from Human Protein Atlas^58^) were considered. For the enrichment analysis, we computed the distribution of genes expressed in a specific brain region among AD-associated genes and compared it with the background distribution of genes expressed in this particular brain region among all the genes that were assayed for expression in any brain regions. Statistically significant expression in a particular brain region was computed using a hypergeometric test (see Methods). No significant enrichment was found for any brain regions among AD-associated genes. This led us to examine these genes from the perspective of anxiety disorder interactomes (ADIs). This framework allowed us to include a larger number of genes in the enrichment analysis and examine AD-associated regions in the context of other mechanistically linked genes.

To assemble the network of PPIs (i.e., interactome) for each type of AD, we collected known PPIs from Human Protein Reference Database (HPRD)^59^ and the Biological General Repository for Interaction Datasets (BioGRID)^60^. The GAD, SAD, PTSD, specific phobia, OCD, and panic disorder interactomes (Supplementary Data S1) were constructed in this manner (see Methods). The ADIs showed significant enrichment in several of the 13 brain regions extracted from GTEx^57^. First, we performed enrichment analyses to determine whether genes in ADIs tend to be overrepresented in a specific brain region. *P *values indicating the statistical significance of the overlap between the interactomes and genes expressed in specific brain regions were computed based on the hypergeometric test (see Methods). *P* value < 0.05 after multiple test adjustments using the Benjamini–Hochberg method was considered to be significant.

**Figure 2:**
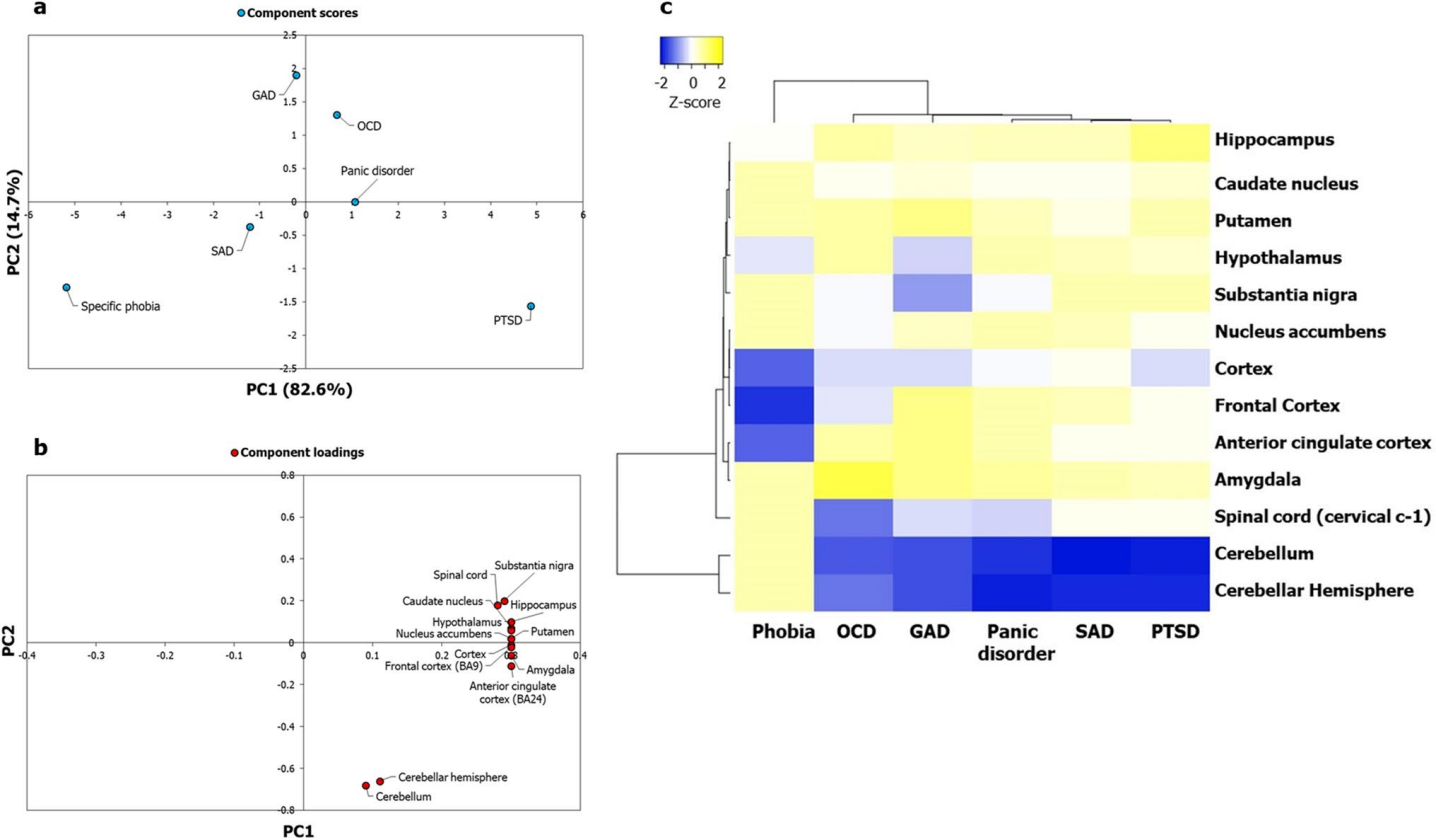
Principal component analysis and clustering analyses of the ADIs based on enrichment patterns in the brain failed to capture putative regional specificities and clusters of the ADs. (**a**) PCA was performed with the *p* values of enrichment of the ADI genes in 13 brain regions compiled from GTEx. *p* values were transformed to − log_10_P values and a data matrix with brain regions (rows) and ADs (columns) (represented as a heatmap in (**c**)) was constructed out of these log-transformed values. Unit variance scaling was applied across this matrix. SVD with imputation was used to extract the principal components (PCs). Component scores (n = 6) corresponding to PC1 and PC2 explaining 82.6% and 14.7% of the total variance were plotted along X and Y axes respectively. (**b**) Component loadings of 13 dimensions, i.e. brain regions, contributing to PC1 and PC2 shown in (**a**) were plotted along X and Y axes respectively. Relatively equal and moderate contribution of all the brain regions, except the cerebellum and the cerebellar hemisphere, shows that ~ 97% of the variance captured by PC1 and PC2 plotted in (**a**) may not have reflected regional specificities of the ADs. (**c**) Variations in region-wise enrichment of ADI genes (computed based on GTEx data) are represented in the form of a heatmap. *p* values indicating statistical significance of enrichment were converted into − log_10_P values. Each cell in the heatmap depicts a normalized z-score derived from a − log_10_P value corresponding to a brain region. Z-scores indicate relative enrichment of specific brain regions in an ADI and are computed based on the number of standard deviations that separate a given *p* value from the mean. Clustering was performed using the hierarchical clustering method with average linkage. The dendrograms were derived from the clustering analysis based on computation of Pearson correlation coefficients between the data points. The region-wise enrichment profile of specific phobia seems to be distinct from that of OCD, GAD, panic disorder, SAD and PTSD, which were all identified to be closely related. Clusters among the latter five disorders were not distinctive. The clustered heatmap was created using Heatmapper (http://www.heatmapper.ca/).

We then sought to understand whether the specific values indicating the significance of enrichment of the ADIs in the brain regions revealed any underlying clustering patterns in terms of regional specificities among the ADs themselves. For this, we generated a data matrix of ADIs (columns) versus brain regions (rows); each cell contained the negative of log-transformed *p* values. Single value decomposition (SVD) with imputation was applied to this matrix to extract principal components that explain the variance observed with region-wise enrichment of gene expression across the ADs. Principal component analysis (PCA) is used to capture systematic variations underlying datasets. Unit variance scaling was applied across the matrix. Six principal components were extracted from the matrix, out of which PC1 and PC2 explained 82.6% and 14.7% of the total variance (Fig. 2a). PTSD and specific phobia seemed to be separated from GAD, OCD, SAD and panic disorders (Fig. 2a). The latter four had low component scores, and clusters among them were not apparent (Fig. 2a). The log-transformed *p* values of enrichment in each brain region were then converted to normalized z-scores. Z-scores indicate the number of standard deviations that separate a given *p* value from the mean. This matrix of z-scores was then subjected to hierarchical clustering based on Pearson’s correlation coefficients and the average linkage method (Fig. 2c). OCD, GAD, panic disorder, SAD and PTSD were depicted as being closely related compared with specific phobia (Fig. 2c). Two factors seemed to differentiate the five ADs from specific phobia: (a) their lower enrichment in the cerebellum and the spinal cord, and (b) their higher enrichment in cortical regions such as the frontal cortex and the ACC (compared with specific phobia).

Next, we sought to identify specific brain regions that were relatively more influential than others in delineating the specific pattern of clustering observed among the ADs. To this end, we examined component loadings that have contributed to PC1 and PC2. Component loadings are values depicting the correlation of the original variables in our data matrix—negative log of *p* value of enrichment for specific brain regions—with each of the extracted principal components. On plotting the component loadings of the brain regions for PC1 and PC2 across X and Y axes, we noticed that almost all of the brain regions were moderately correlated with PC1 and PC2 and contributed relatively equally to them (Fig. 2b). Hence, although the PC plot of ADs explained ~ 97% of the variance observed in region-wise enrichment (Fig. 2b), it may not have captured region specificities underlying the ADs, except for the ability of cerebellar structures, spinal cord and cortical regions to differentiate specific phobia from OCD, GAD, panic disorder, SAD and PTSD. The same pattern of moderate correlation of most of the component loadings with PC1 and PC2 and their equivalent contribution to the principal components was observed with a larger set of 26 brain regions extracted from Allen Brain Atlas (genes that are not housekeeping genes and have logRPKM > 2, where RPKM is Reads Per Kilobase per Million mapped reads, were considered) (Supplementary Fig. S1).

### Potential striatal association of ADIs

We checked the overlap among the six ADIs. Thirty-six genes were found to be shared among all the interactomes (Supplementary Table S2). These genes were found to be enriched only in striatal genes, i.e., genes with high/medium expression in the caudate nucleus, putamen and the nucleus accumbens (all at *p* value = 0.0187) (based on data extracted from GTEx), suggesting a potential relationship between ADs and striatal gene dysfunction.

Early developmental mechanisms controlled by genetic susceptibility factors and gene-environment interactions may modulate the response pattern of an individual to threat stimuli^5^. Genes involved in establishing neuronal connectivity in the adult brain are often regulated by genes that specify the neuronal identity and brain regionalization during the early stages of brain development. We reasoned that the striatum-enriched genes that co-occur in all the ADs could be closely connected with genes that set up the cellular and molecular architecture of the striatum during early developmental stages, namely, transcriptional regulators and signaling transduction molecules controlling neuronal development and neurotransmission^61^. Specifically, these genes are DLX1-6, GSX2, EBF1, ISL1, FOXP1/2, DRD1/2, GNAL, ADCY5, PPP1R1B, STEP and RASGRP2^61^. We indeed observed that these genes involved in striatal development were closely interconnected with our striatum-enriched genes (Supplementary Fig. S2). Several anxiety-associated genes had direct interactions with striatal development genes, such as PRKCA with ADCY5 and DLX3, and ESR1 with ISL1 (Supplementary Fig. S2). We found this network to be enriched in dorsal thalamus genes (*p* value = 0.0143) in addition to striatal genes (*p* value = 0.0016) (based on data extracted from Allen Brain Atlas). These results imply that the striatum is critical for ADs from the perspective of their interactomes.

### Distinctive overlaps of ADIs and SMIs

Striatum is composed of two neurochemically segregated compartments called the striosomes and the matrix, which are characterized by their distinct gene expression profiles^47^. We analyzed these structures from an interactome perspective. We compiled the list of genes differentially expressed in the striosomes and the matrix compartments (Supplementary Table S3) of various species (rat, mouse, ferret, cat, monkey and human)^47^, and mapped them to their human orthologs. From enrichment analysis using Gene Ontology terms, we noted that some genes differentially expressed in the striosomes (HTR2A, HTR2C, CHRM1-5 and DRD4) were involved in the serotonergic signaling pathway (*p* value = 2.12E−10); human striosomes are known to be enriched in serotonin receptors^62^. Some genes differentially expressed in the matrix neurons (CDH8, CDK5, CNR1, HTR2A and SLC17A6) were found to be involved in the glutamatergic synaptic transmission (*p* value = 1.93E−5). We examined whether the interactome of the genes differentially expressed in either of these striatal compartments were significantly enriched in each of the ADIs.

**Figure 3:**
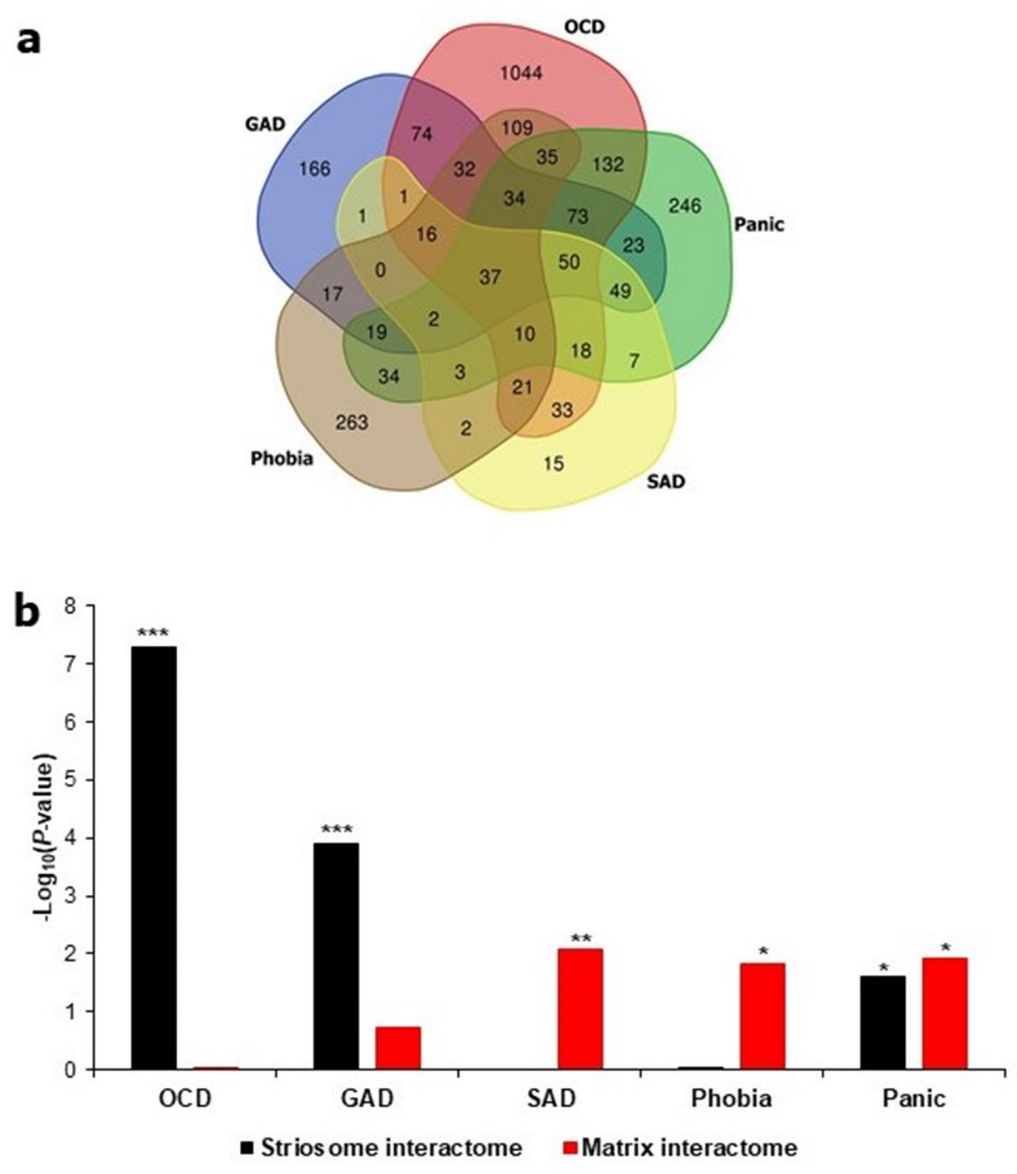
ADIs showed preferential and statistically significant overlaps with either SI or MI. (**a**) The number of proteins shared among ADIs is shown as a Venn diagram. (**b**) The number of common proteins shared between a particular ADI and SMIs assembled from the genes differentially expressed in striosome/matrix was computed. − log_10_P values derived from this analysis are shown as bars in the figure. Overlaps with *p* value < 0.05 (i.e. − log_10_P > 1.3) after correction for multiple hypotheses were considered to be statistically significant. *, ** and *** corresponds to *p* value < 0.05, *p* value < 0.01 and *p* value < 0.001 respectively. Four out of the 5 ADIs shown in the figure shared statistically significant and exclusive overlaps with either the SI (OCD and GAD) or the MI (SAD and phobia). While the panic disorder interactome shared statistically significant overlaps with both of the striatal compartments, the overlap shared with the MI was more statistically significant than that shared with the SI. From (**a**), it is clear that the ADIs themselves do not segregate into any groups. However, (**b**) shows that they exhibit preferential overlap with SMIs. For example, (**a**) shows that the OCD interactome shares 186 genes with the SAD interactome, whereas (**b**) clarifies that the OCD and SAD interactomes exhibit preferential overlaps with the striosome and MIs respectively. The Venn diagram tool provided as part of the Bioinformatics & Evolutionary Genomics toolkit was used to create the Venn diagram (http://bioinformatics.psb.ugent.be/webtools/Venn/).

Computation of overlaps among the ADIs revealed that they themselves do not segregate into any groups (Fig. 3a). However, specific ADIs showed significant and distinctive overlaps (Fig. 3b and Table 1) with the striosome/matrix interactomes (SMIs) (Supplementary Data S2). The striosome interactome (SI) shared 28% of its constituent genes with the GAD interactome (227 genes out of a total of 810 SI genes) (*p* value = 1.31E−4) (i.e. out of the total number of 810 genes present in SI, 227 genes were also found in the GAD interactome) and 53% (427/810) of genes with the OCD interactome (*p* value = 5.21E−8). These genes shared with the SI account for 38% (227/595) and 25% (427/1718) of the genes in the GAD and OCD interactomes, respectively. Out of the 1718 genes in the OCD interactome, 639 were not shared with any other ADI. 17.5% of these 639 (112/639) genes were shared with the SI (*p* value = 2.27E−7) (overlap between OCD and SI is illustrated in the form of a network diagram in Supplementary Fig. S3). Genes shared between OCD and SI showed high enrichment for genes associated with human motor and behavioral stereotypes (Human Phenotype Ontology^63^) (Supplementary Note S1). This observation is in line with the findings of a study that demonstrated the ability of excessive activation in the striosomes (compared with the matrix) to predict the degree of drug-induced motor stereotypy in rats^54^; both the activation ratio and drug-induced stereotypy have been shown to be under the regulation of cholinergic interneurons in the striatum^64–66^. The matrix interactome (MI) shared 14.35% (30/209) of its constituent genes with the SAD interactome (*p* value = 8.23E−3). The MI shared 23% (48/209) of its constituent genes with the phobia interactome (*p* value = 0.0154). The genes shared with the MI account for 11% (30/266) and 7.5% (48/634) of the genes in the SAD and phobia interactomes. 32.5% (68/209, *p* value = 0.012) and 26% (212/810, *p* value = 0.025) of genes found in MI and SI respectively were also found in the panic disorder interactome. In addition to striatum-expressed genes, thalamus-expressed genes were enriched in the network of striatal developmental regulators and anxiety-associated genes (Supplementary Fig. S2). Unlike striatal sub-compartments, molecularly distinct subdivisions of the thalamus did not show any preferential association with any ADI (Supplementary Methods and Supplementary Note S2).

**Table 1:** Overlap of the anxiety disorder and striatal subcompartment interactomes. The table shows the statistics of the overlaps shared between the five anxiety disorder interactomes and the interactomes of the striatal subdivisions.

Overlap between Interactomes and the *P *value of significance	Strisome Interactome (810 genes)	Matrix Interactome (209 genes)
Generalized anxiety disorder interactome (595 genes)	227 (1.31E−04)	n.s
Obsessive compulsive disorder interactome (1718 genes)	427 (5.21E−08)	n.s
Panic disorder interactome (773 genes)	212 (0.025)	68 (0.012)
Social anxiety disorder interactome (266 genes)	n.s	30 (8.23E−03)
Specific phobia interactome (634 genes)	n.s	48 (0.0154)

In summary, each ADI shared a statistically significant overlap with SMIs, i.e., the GAD interactome shared an overlap with the SI, OCD with SI, phobia with MI and SAD with MI. Although panic disorder showed significant overlaps with SI and MI, the statistical significance of the overlap with MI was higher than that of the overlap with SI. These results raised the possibility that gene dysfunction occurring in striosomes could underlie the symptoms of GAD and OCD, whereas matrix dysfunction could underlie SAD and phobia. Hence, five groups of shared genes contributing to statistically significant overlaps of ADIs with SMIs (referred to as ‘AD-SMIs’ henceforth) were delineated from this analysis, namely, GAD-striosome, OCD-striosome, SAD-matrix, phobia-matrix and panic-matrix. We focused on these five AD-SMIs speculating that they would allow us to probe the etiological differentiation of these ADs in terms of their affiliation to one of the two striatal compartments.

### Expression of AD-SMI genes in brain regions

Studies have noted a substantial overlap between functional connectivity and gene co-expression patterns within and between cortical and striatal networks^18–23^. Functional connectivity can be defined as a temporal correlation between brain regions, often derived from co-activated fMRI signals at resting state^67^. Co-expression patterns are derived from Pearson and Spearman correlations that assess the transcriptional similarity of genes. Based on this, we speculated that testing the regional expression patterns of AD-SMIs may reveal brain regions showing expression of these shared genes and perhaps, exhibiting functional connectivity with the striatal compartments and governing key anxiety traits. However, it is important to note that (a) the validity of this speculation is supported only for a limited number of brain networks, and (b) the degree and the nature of the interaction between transcriptional similarity and functional connectivity are yet to be fully elucidated^23^. We thus examined the expression patterns of AD-SMIs (e.g., genes shared between GAD interactome and SI) in the brain.

**Figure 4:**
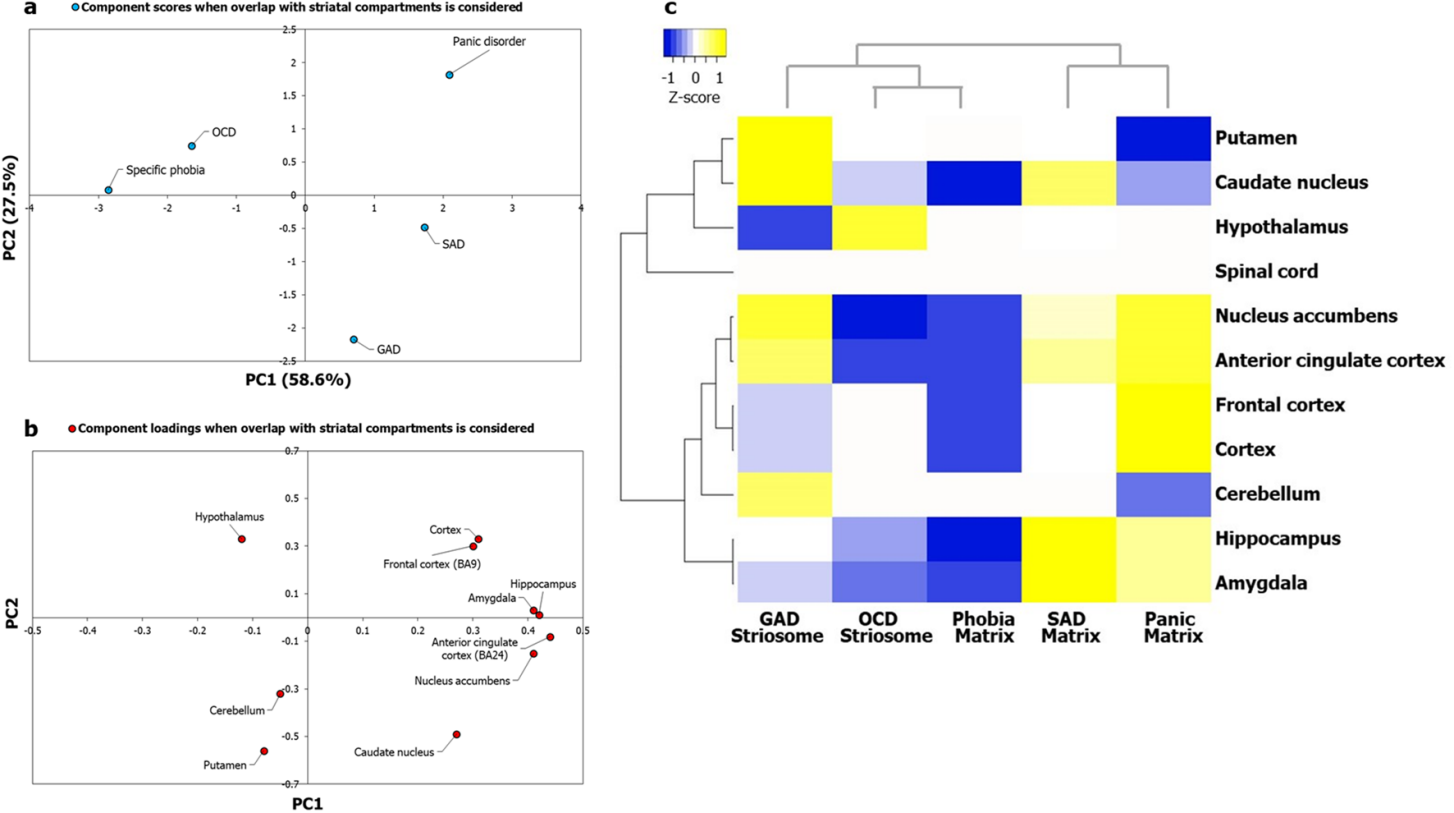
Principal component analysis and clustering analyses of AD-SMIs revealed uneven influence of key brain regions linked to anxiety etiology and distinct AD groups. (**a**) PCA was performed with the *p* values of enrichment of genes that co-occur in specific ADIs and SMIs. As observed in Fig. 5, specific ADIs share discrete and distinct statistically significant overlaps with either SI or MI, namely, GAD and striosome, OCD and striosome, phobia and matrix, SAD and matrix, and panic disorder and matrix. The enrichment of these genes among those exhibiting high/medium expression in 13 brain regions (TPM > 9) compiled from GTEx was checked. The statistical significance of region-wise enrichment was computed as *p* values. These values were transformed to − log_10_P values, which were then assembled into a data matrix containing brain regions as rows and ADs as columns (represented as a heatmap in Fig. (**c**)). Unit variance scaling was applied across this matrix. Single value decomposition (SVD) with imputation was used to extract the principal components (PCs). Component scores of GAD-striosome, OCD-striosome, phobia-matrix, SAD-matrix and panic disorder-matrix (n = 5) corresponding to PC1 and PC2 explaining 58.6% and 27.5% of the total variance were plotted along X and Y axes respectively. (**b**) Component loadings of 10 dimensions, i.e. brain regions, contributing to PC1 and PC2 shown in (**a**) were plotted along X and Y axes respectively. In contrast with the pattern of component loadings of brain regions observed in Fig. 2b, PCA with genes shared between ADIs and SMIs appears to have captured uneven influences of several brain regions such as the ACC, amygdala, hippocampus, nucleus accumbens, putamen and caudate on the grouping patterns of ADs. (**c**) Variations in region-wise enrichment of genes (computed from GTEx data) shared between ADIs and SMIs are represented in the form of a heatmap. Specifically, normalized z-scores computed based on the –log_10_ transformed *p* values, indicating the statistical significance of enrichment of GAD-striosome, OCD-striosome, phobia-matrix, SAD-matrix and panic disorder-matrix, are shown in the figure. Z-scores indicate relative enrichment of specific brain regions in the gene sets and are computed based on the number of standard deviations that separate a given *p* value from the mean. Clustering was performed using the hierarchical clustering method with average linkage. The dendrograms were derived from the clustering analysis based on computation of Pearson correlation coefficients between the data points. Two main clusters were detected among the ADs. SAD and panic formed one cluster. OCD and phobia formed a sub-cluster within the second main cluster. GAD was an outgroup to the sub-cluster of OCD and phobia. The clustered heatmap was created using Heatmapper (http://www.heatmapper.ca/).

Lists of genes expressed in 13 postnatal human brain regions were extracted from GTEx^57^, and their enrichment in each of the five AD-SMIs was systematically computed using the hypergeometric test (see Methods). Multiple brain regions showed significant enrichment in each of these groups. Following this, we used the negative of log-transformed *p* values denoting the significance levels of enrichment as input data for PCA. Five principal components were extracted, and from this, we selected PC1 and PC2, explaining 58.6% and 27.5% of the variance observed with region-wise enrichment in gene expression to interpret the AD groupings. Notably, we observed that OCD, GAD, SAD, and panic disorder exhibited clearer patterns of clustering when the overlap with striatal compartments was taken into consideration (Fig. 4a) compared to when this overlap was not taken into consideration (Fig. 2a). Next, we generated a heat map of z-scores derived from the log-transformed *p* values and employed Pearson correlation and average linkage method to identify clusters of ADs. Firstly, we observed two main clusters (Fig. 4c): the first cluster included SAD-matrix and panic-matrix, and the second cluster included GAD-striosome and a sub-cluster consisting of OCD-striosome and phobia-matrix. The occurrence of GAD-striosome and OCD-striosome in the same cluster (Fig. 4c) is supported by previous observations linking striatal stimulation and striatal beta oscillation to a key feature in OCD called cognitive inflexibility, which manifested as a repetitive pattern of a major symptom observed in GAD called pessimistic valuation^50^. Secondly, with the introduction of striatal subdivisions in our analysis with AD-SMIs (Fig. 4c), we were able to delineate the clustering of caudate nucleus and putamen, and nucleus accumbens and ACC, a pattern that was not clear with ADIs (Fig. 2c). This demonstrates the biological validity of our approach. Additionally, it is notable that the spinal cord did not influence the clustering of the AD-SMIs (Fig. 4c).

**Figure 5:**
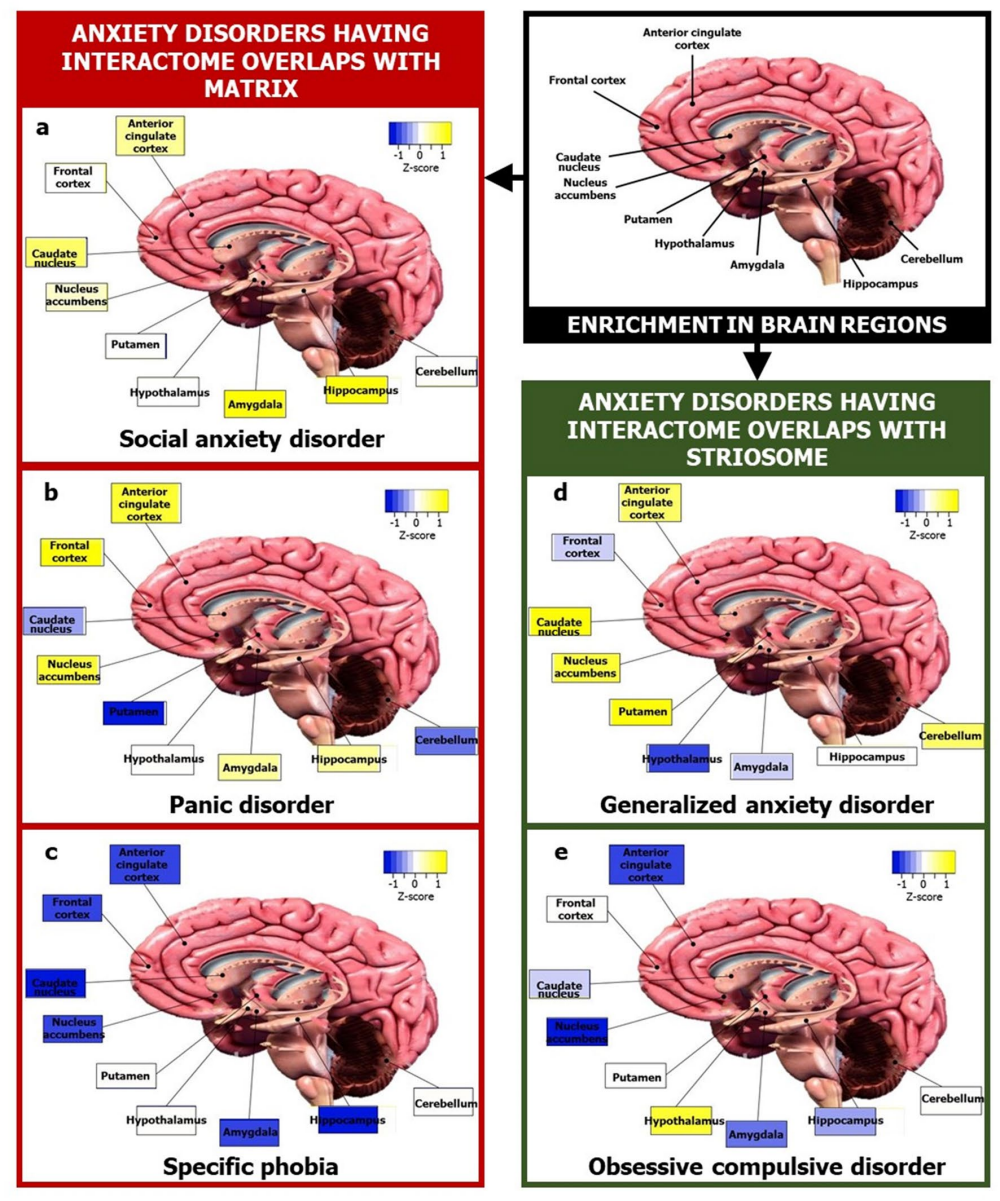
Map of focal brain regions in various ADs. The figure shows brain regions that may be linked to the etiology of two categories of ADs, namely, those with striosomal (green boxes) affiliation and those with matrix (red boxes) affiliation, in terms of interactome overlaps. The regions in the ‘brain map’ of each AD was colored according to the normalized z-score for that region indicating its mean enrichment in the ADI. This was computed using data from GTEx as shown in Fig. 4c. Prominent involvement of ACC in generalized anxiety disorder (**d**) and that of amygdala and hippocampus in social anxiety disorder (**a**) can be noted. The brain section image is a royalty-free stock illustration (ID: 1401181217) downloaded from Shutterstock titled ‘Human Brain Anatomy Sagittal Section with Labels, 3D Rendering’.

Further, we plotted the correlation of the component loadings with PC1 and PC2 to assess whether the observed pattern of clustering reflected regional specificities (Fig. 4b). It was observed that some brain regions had an uneven influence over the clustering pattern when overlap with striatal compartments was taken into consideration (Fig. 4b), namely, ACC, amygdala, hippocampus, nucleus accumbens, putamen and caudate, compared to when the striatal overlap was not taken into consideration (Fig. 2b). Heightened reactivity in the first three regions has been associated with clinical anxiety^68^. We identified two groups of brain regions that may more or less act as functional units to influence the etiology of these two purported ‘types’ of ADs (Fig. 4c): (1) amygdala and hippocampus and (2) ACC and nucleus accumbens. They are referred to here as ‘functional units’ by virtue of them being tight clusters in the dendrogram, and only in terms of their potential contribution towards anxiety etiology (see Supplementary Discussion). Higher enrichment of SAD-matrix and panic-matrix in the amygdala and hippocampus may have segregated them from the cluster of GAD-striosome, OCD-striosome and phobia-matrix that showed lower enrichment in these same regions (Fig. 4c). Relatively higher enrichment of GAD-striosome in ACC and nucleus accumbens may have led to its segregation from the sub-cluster of OCD-striosome and phobia-matrix (Fig. 4c). Based on this analysis, we constructed ‘brain maps’ for two categories of ADs, namely, those sharing interactome overlaps with striosome and with matrix (Fig. 5).

**Figure 6:**
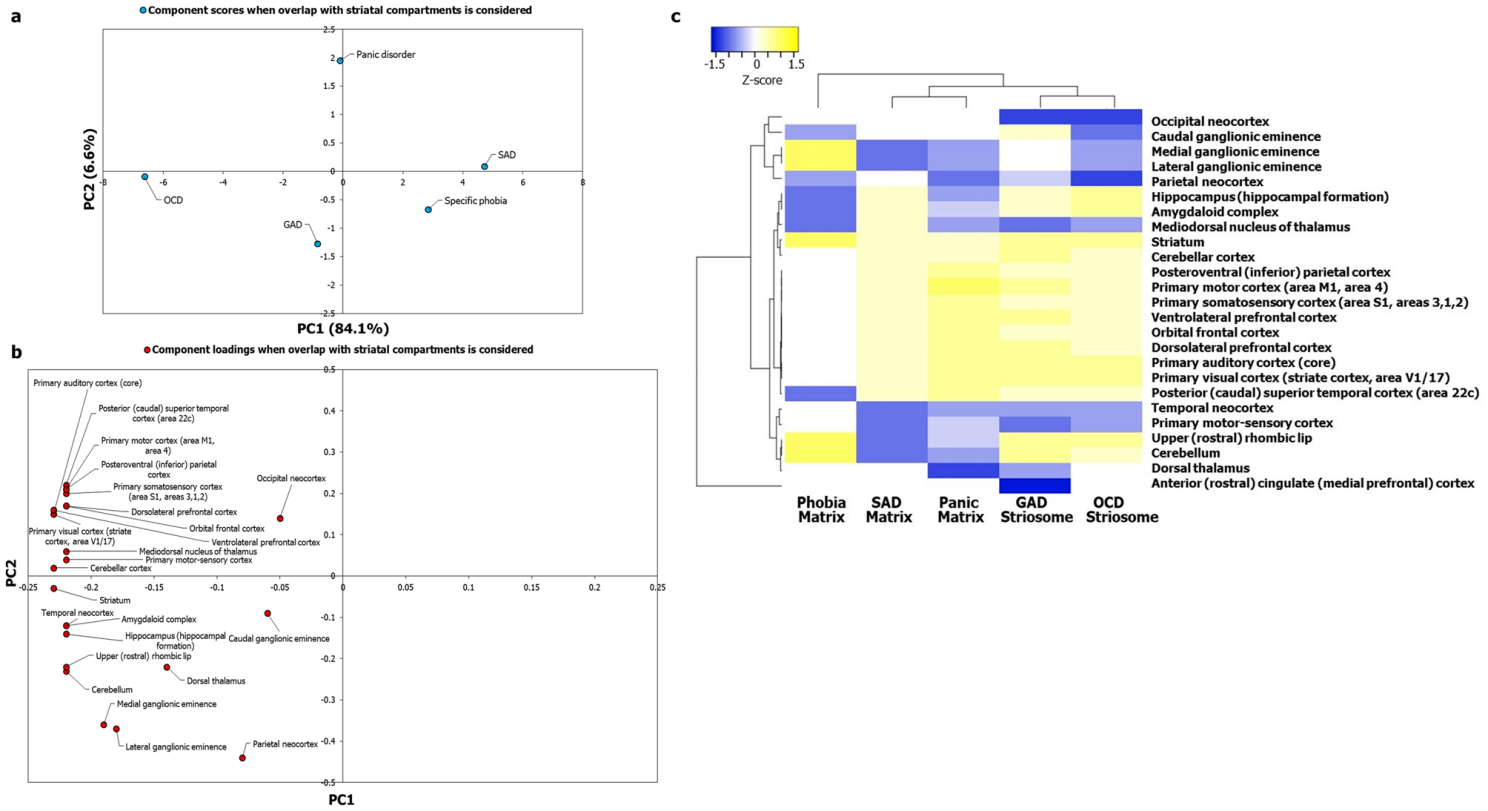
Principal component analysis and clustering analyses of AD-SMIs based on a larger expression dataset recapitulated the AD groups and revealed embryonic structures potentially relevant to anxiety etiology. (**a**) PCA was performed with the *p* values of enrichment of genes that co-occur in specific ADIs and SMIs. The enrichment of these genes among those expressed across 26 brain regions compiled from Allen Brain Atlas having log(RPKM) > 2 was checked. The statistical significance of region-wise enrichment was computed as *p* values. These values were transformed to − log_10_P values, and then assembled into a data matrix containing brain regions as rows and ADs as columns (represented as a heatmap in (**c**)). Unit variance scaling was applied across this matrix. Single value decomposition (SVD) with imputation was used to extract the principal components (PCs). Component scores of GAD-striosome, OCD-striosome, phobia-matrix, SAD-matrix and panic disorder-matrix (n = 5) corresponding to PC1 and PC2 explaining 84.1% and 6.6% of the total variance were plotted along X and Y axes, respectively. (**b**) Component loadings of 24 dimensions, i.e., brain regions, contributing to PC1 and PC2 shown in (**a**) were plotted along X and Y axes, respectively. This figure recapitulates findings from GTEx data (Fig. 4c) with data from Allen Brain Atlas, namely, observation of clearer grouping patterns among ADs when the striatal overlap is taken into consideration and uneven contribution of multiple brain regions to these patterns such as medial ganglionic eminence, lateral ganglionic eminence and parietal cortex. (**c**) Variations in region-wise enrichment of genes between ADIs and SMIs are represented in the form of a heatmap. Specifically, normalized z–scores computed based on the –log_10_ transformed *p* values, indicating the statistical significance of enrichment of GAD-striosome, OCD-striosome, phobia-matrix, SAD-matrix and panic disorder-matrix, are shown in the figure. Z-scores indicate relative enrichment of specific brain regions in the gene sets and are computed based on the number of standard deviations that separate a given *p* value from the mean. Clustering was performed using the hierarchical clustering method with average linkage. The dendrograms were derived from the clustering analysis based on the computation of Pearson correlation coefficients between the data points. Clustering of GAD with OCD and SAD with panic disorder seen with GTEx data in Fig. 4c is recapitulated here. Phobia was identified as a separate cluster altogether. The clustered heatmap was created using Heatmapper (http://www.heatmapper.ca/).

We examined whether the AD-SMIs were enriched for expression in 23 postnatal human brain regions and three transitory fetal structures (lateral ganglionic eminence, medial ganglionic eminence and rhombic lip) available in Allen Brain Atlas^69^. PCA showed that the clustering among the ADs was clearer when overlap with the striatal compartments was taken into consideration (Fig. 6a) compared with when this overlap was not taken into consideration (Supplementary Fig. S1a). Despite using a more diverse and numerous dataset, hierarchical clustering revealed the preservation of the grouping of GAD-striosome with OCD-striosome and SAD-matrix with panic-matrix, both of which appeared as sub-clusters within the main cluster in this analysis; phobia was detected as an outgroup to this main cluster (Fig. 6c). Two groups of brain structures seemed to be highly influential in this clustering (Fig. 6c): (1) medial ganglionic eminence (MGE) and lateral ganglionic eminence (LGE) and (2) parietal neocortex. LGE is a source of striatal projection neurons and gives rise to both striosomes and matrix neurons^70^. MGE populates cortical layers and differentiates into interneurons^71^. The segregation of the sub-clusters of GAD-striosome and OCD-striosome from SAD-matrix and panic-matrix could have stemmed from the higher enrichment of LGE and MGE in the former group compared with the latter group (Fig. 6c). It was interesting to note that phobia-matrix showed high enrichment for LGE and MGE similar to GAD-striosome and OCD-striosome (Fig. 6c), which may explain their occurrence in the same cluster in Fig. 4c and hint at shared etiology rooted in perturbations of genes expressed during the early stages of striosome-matrix compartment specification. Phobia-matrix had a lower enrichment for parietal cortex (and CGE which clustered together) compared to GAD-striosome and OCD-striosome. GAD-striosome showed exclusive enrichment for ACC (Fig. 6c). Lastly, we examined the correlation of component loadings with PC1 and PC2, which captured 90.7% of the variance observed in region-wise enrichment of gene expression. This ascertained the fact that the observed pattern of clustering was unevenly influenced by regional specificities that become apparent when overlap with striatal compartments is taken into consideration (Fig. 6b). These regional specificities were not observed when the striatal overlap was not considered (Supplementary Fig. S1b). From this detailed analysis, we can confirm that regional specificities underlying the various ADs are revealed only when discrete subnetworks of their interactomes that contain genes differentially expressed in the striosome/matrix and their interactors are examined. Since functional connectivity and gene expression are correlated in the brain^18–23^, this raises the possibility that the etiology of specific ADs may be rooted in the discrete functional connections of specific striatal sub-compartments with other brain regions, such as the ACC and amygdala, that govern traits specific to these ADs.

### Signaling pathways enriched in key brain regions influential in the grouping of ADs

**Figure 7:**
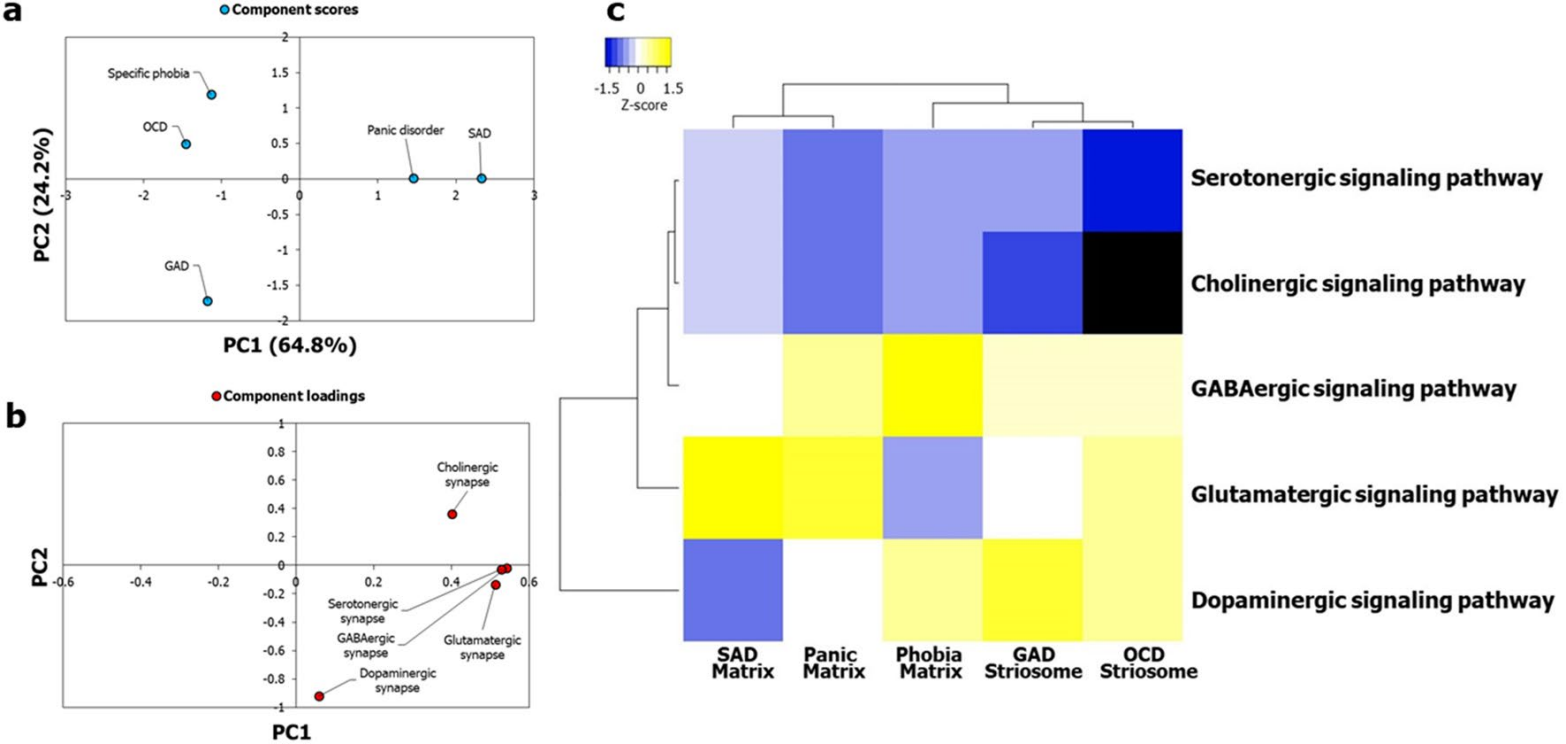
Neuronal synaptic signaling pathways enriched among genes expressed in influential brain regions recapitulated the AD groups. Gene sets in GAD-striosome, OCD-striosome, phobia-matrix, SAD-matrix and panic-matrix showing moderate/high expression in the four key brain regions (i.e., amygdala, hippocampus, ACC and nucleus accumbens) were selected. The KEGG pathways enriched in them were then identified. (**a**) PCA was performed with the *p* values of enrichment of five neuronal synaptic signaling pathways in each of these gene sets, namely, cholinergic, dopaminergic, GABAergic, glutamatergic and serotonergic signaling pathways. These values were transformed to –log_10_P values and were then assembled into a data matrix containing pathways as rows and the gene sets as columns (represented as a heatmap in (**c**)). Unit variance scaling was applied across this matrix. Single value decomposition (SVD) with imputation was used to extract the principal components (PCs). Component scores of gene sets from GAD-striosome, OCD-striosome, phobia-matrix, SAD-matrix and panic disorder-matrix (n = 5) corresponding to PC1 and PC2 explaining 64.8% and 24.2% of the total variance were plotted along X and Y axes respectively. Genes in GAD-striosome, OCD-striosome, phobia-matrix, SAD-matrix and panic-matrix that were involved in at least one of the five neuronal synaptic signaling pathways, and showed expression in at least one of the four brain regions that were influential in producing the pattern seen in Fig. 4a,c, recapitulated the AD groups seen in Fig. 4a,c. (**b**) Component loadings of 5 dimensions, i.e. signaling pathways, contributing to PC1 and PC2 shown in (**a**) were plotted along X and Y axes respectively. The loading value of the dopaminergic signaling pathway indicated that it had high influence over the observed grouping pattern. GABAergic and glutamatergic signaling pathways seemed to have a moderate influence. (**c**) Pathway enrichment of these gene sets are represented in the form of a heatmap. Specifically, normalized z-scores computed based on the –log_10_ transformed *p* values, indicating the statistical significance of pathway enrichment of these gene sets, are shown in the figure. The dendrograms were derived from hierarchical clustering analysis based on the computation of Pearson correlation coefficients between the data points. The clustered heatmap was created using Heatmapper (http://www.heatmapper.ca/).

We isolated the gene sets that were responsible for the enrichment of the AD-SMIs in the four brain regions—amygdala, hippocampus, ACC and nucleus accumbens—that were presumably more influential than the other brain regions (Fig. 4b) in producing the grouping pattern seen in Fig. 4a,c, i.e., the genes that were responsible for the *p* values of enrichment used for generating Fig. 4a and Fig. 4c. For example, the gene set for GAD-striosome contained the genes shared between GAD-striosome and the four brain regions, i.e., the genes which co-occurred in GAD interactome and SI, and showed expression in at least one of the four brain regions. For a particular AD-SMI, the same set of genes was found to be expressed across the four brain regions, which led us to consider them together in the corresponding gene set. The signaling pathways (KEGG^72^) that were significantly enriched (*p* value after multiple test adjustment < 0.05) in each of these gene sets were identified using WebGestalt^73^. A data matrix of the enriched pathways (rows) and disorder-striatal compartment combinations (columns) was created; each cell contained −log_10_P of enrichment of each pathway. Firstly, on PCA and clustering analysis of this matrix, we observed that five neuronal synaptic signaling pathways, namely, the cholinergic, dopaminergic, GABAergic, glutamatergic and serotonergic signaling pathways, could produce a grouping pattern (Fig. 7a, c) similar to that shown in Fig. 4a and Fig. 4c. Out of these, the dopaminergic signaling pathway had a very high influence on the grouping pattern, and the GABAergic and glutamatergic signaling pathways had a moderate influence (Fig. 7b). Based on this, we speculated that the conservative set of genes co-occurring in each of the AD-SMIs and expressed in the four connected limbic structures are those that influence dopamine signaling. Secondly, we noted that when all the enriched pathways were considered, phobia-matrix clustered alongside SAD-matrix and panic-matrix (Supplementary Fig. S4), and not with GAD-striosome and OCD-striosome.

**Figure 8:**
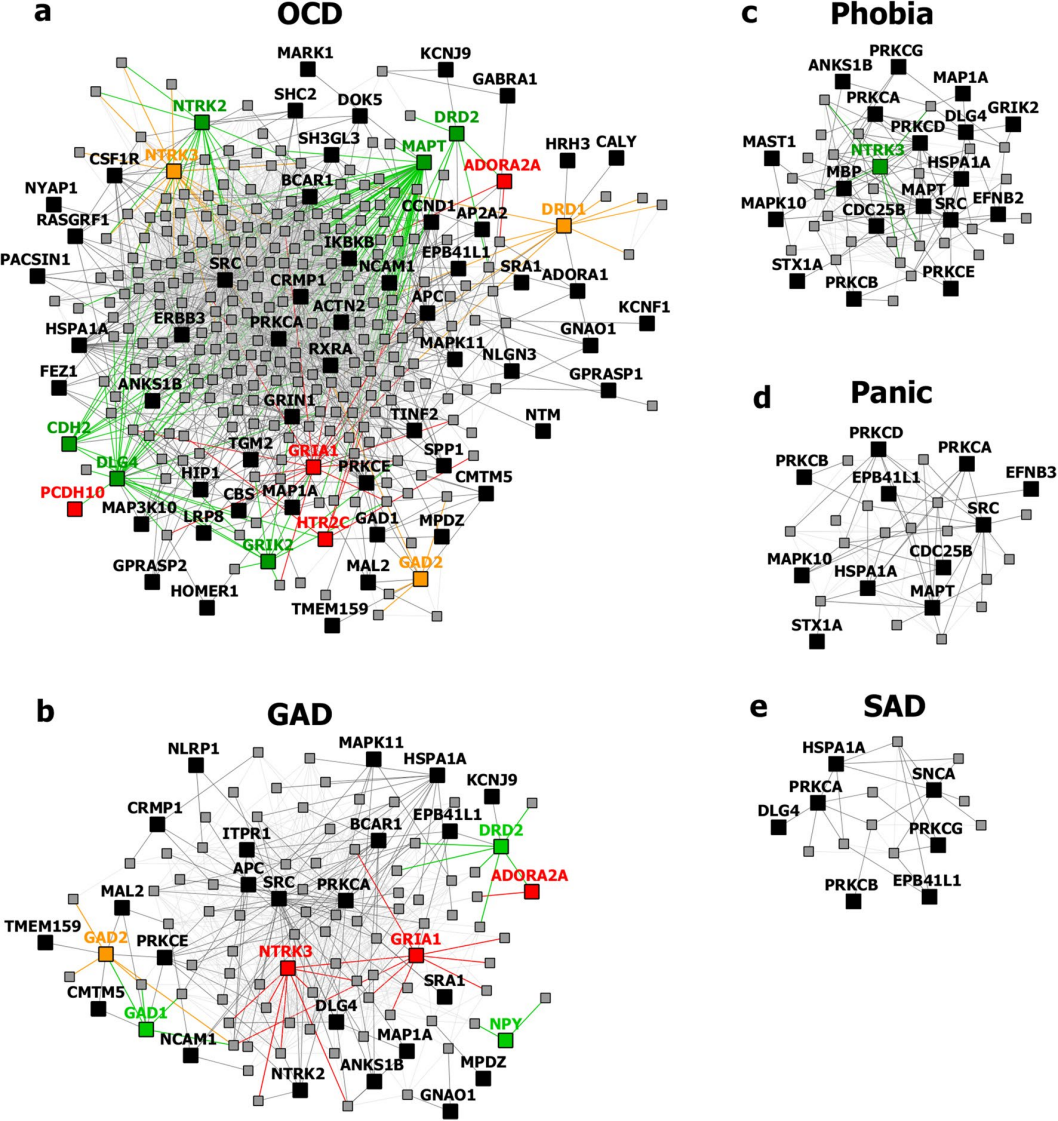
Network layout of the genes expressed in the influential brain regions in the AD-SMIs. The network diagrams show the genes having moderate/high expression in the four brain regions that were highly influential in identifying the disorder groups shown in Fig. 4c, namely, amygdala, hippocampus, ACC and nucleus accumbens. These genes have been highlighted in (**a**) OCD interactome, (**b**) GAD interactome, (**c**) phobia interactome, (**d**) panic disorder interactome and (**e**) SAD interactome. In each of the network diagrams, green colored nodes depict genes associated with the particular AD. Red colored nodes indicate genes differentially expressed in striosomes/matrix that shows interactome overlap with the given AD; (**a**) OCD and (**b**) GAD shows interactome overlap with striosome, whereas (**c**) phobia, (**d**) panic disorder and (**e**) SAD show overlap with matrix. Orange colored nodes indicate genes that are associated with the particular AD as well as differentially expressed in the striatal compartment. Black colored nodes are intermediary genes interconnecting the AD genes. The majority of the red and black colored nodes shown in the diagrams, i.e. genes that are yet to be studied in the context of human ADs, have already been linked to anxiety-like/repetitive/perturbed social behavior in animal models and humans (see Table 2). The validity of these genes as candidates for further investigation of anxiety mechanisms is supported by this evidence and their proximity in the interactome to genes that have been previously associated with human ADs (i.e., green colored nodes). The network diagrams were created using Cytoscape (version 3.7.2).

Collectively, the gene sets that were examined, as discussed above, contained 84 genes (Fig. 8). Out of these, 12 were known to be associated with ADs (CDH2, DLG4, DRD1, GRIK2, MAPT and NTRK2 associated with OCD, NTRK3 with OCD and panic disorder, ADORA2A with panic disorder and specific phobia, GAD1 with GAD and panic disorder, NPY with GAD, panic disorder and specific phobia, GAD2 with GAD, OCD and panic disorder, and DRD2 with GAD, OCD, SAD and specific phobia). These genes were used as starting points for interactome construction in our study. The rest of the 72 genes, including three genes that were differentially expressed in striosomes/matrix (GRIA2, HTR2C and PCDH10)^47^, have not been studied so far in the context of human ADs. However, 59 (82%) of these genes have already been linked to anxiety-like/linked behavior/physiological states, repetitive behavior or perturbed social behavior through gene knockout, differential gene expression/methylation studies in animal models and genetic variant studies in humans (see evidence from each of these studies in Table 2). This demonstrates the validity of our interactome-based method due to two reasons: we (a) identified other genes that were previously studied in animal models of anxiety using an unbiased approach from the ADIs, and (b) showed that they were closely connected to the genes associated with human ADs, thereby providing a mechanistic framework to study these putative anxiety-associated genes in the future.

**Table 2:**
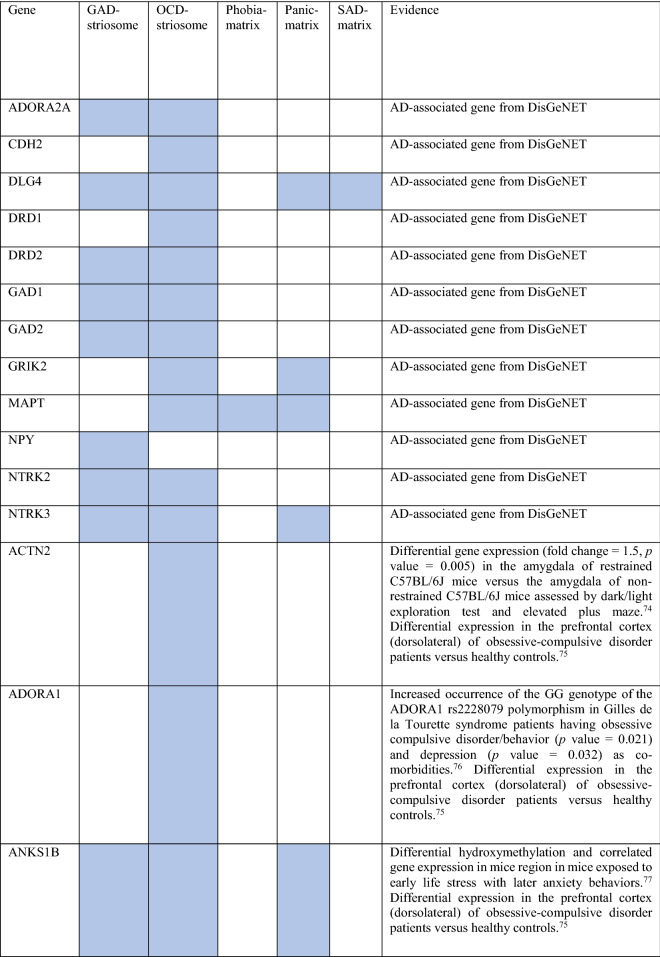

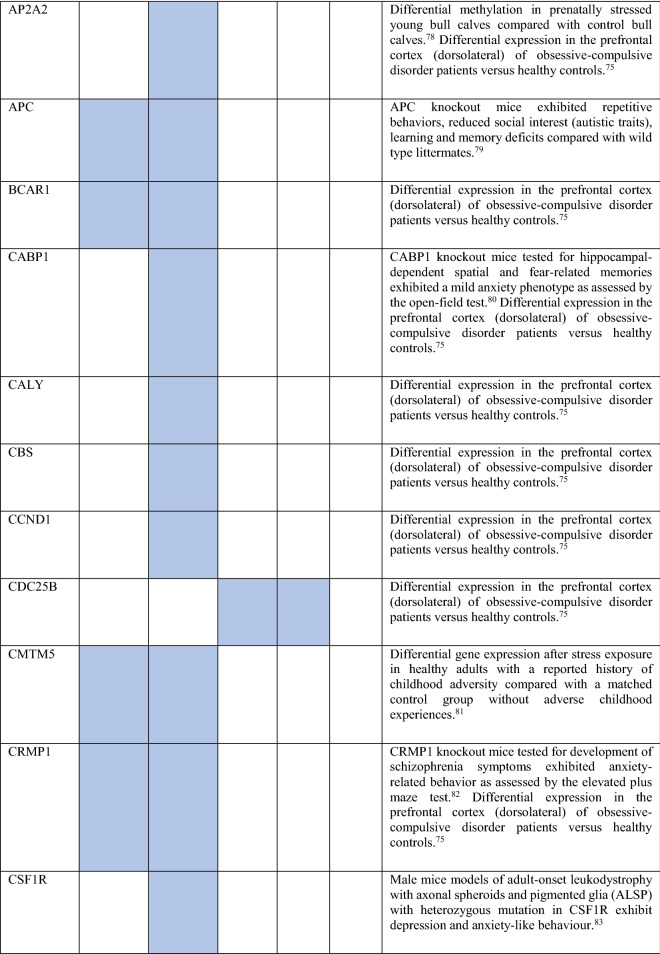

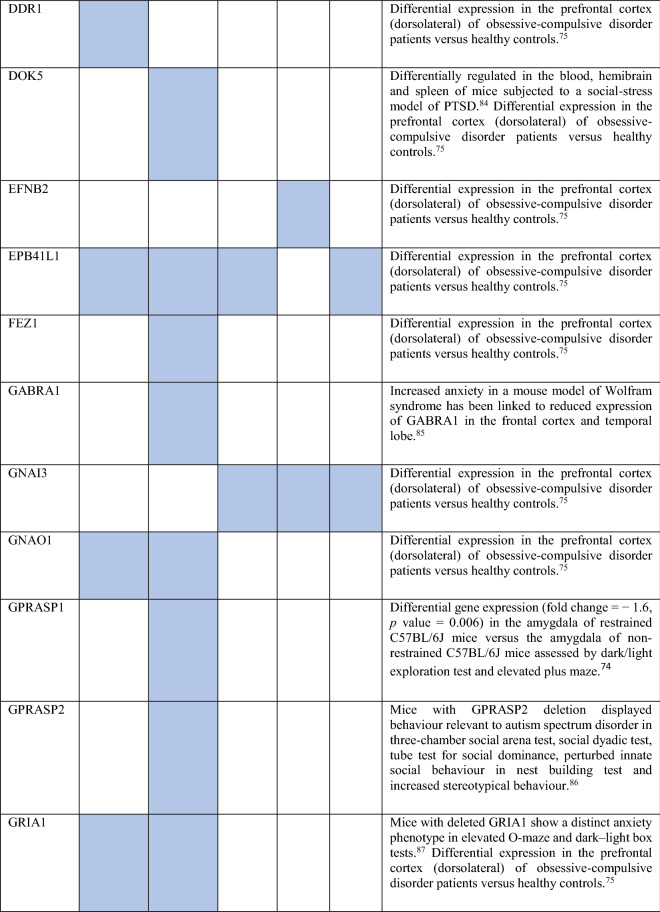

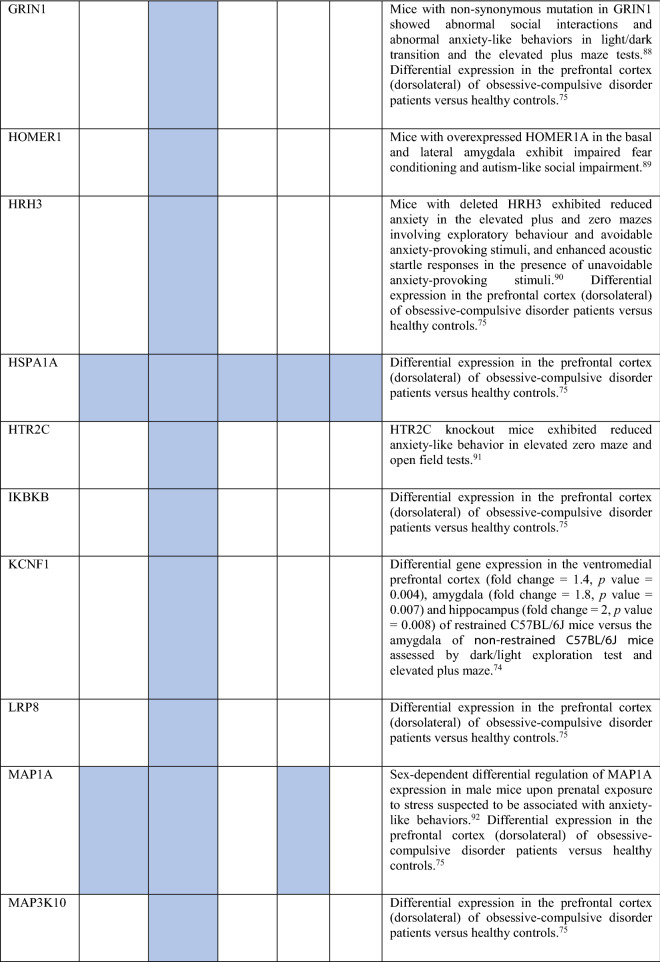

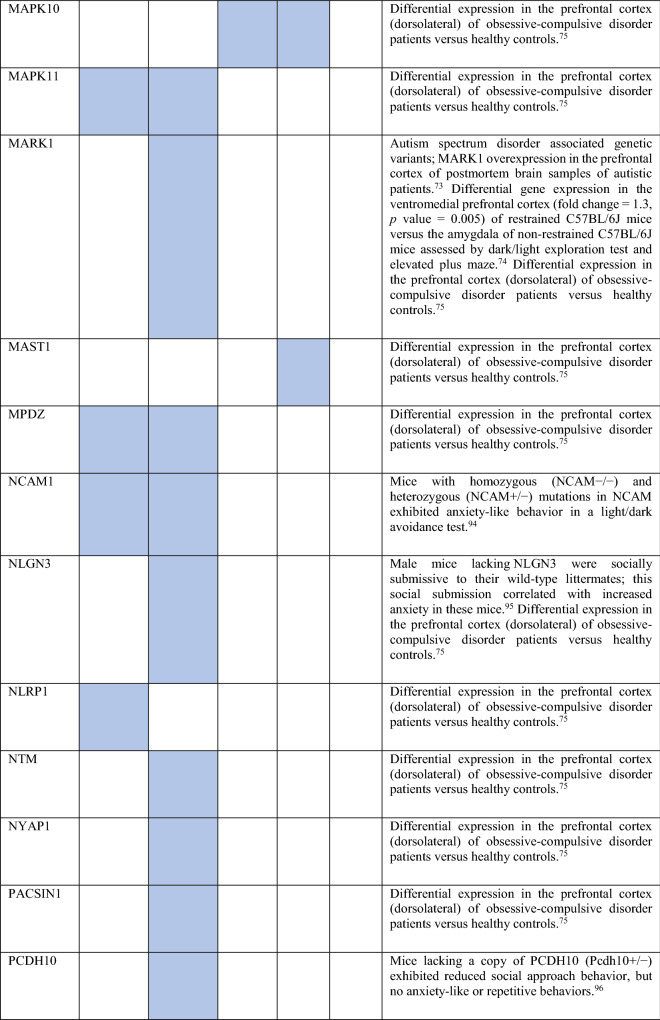

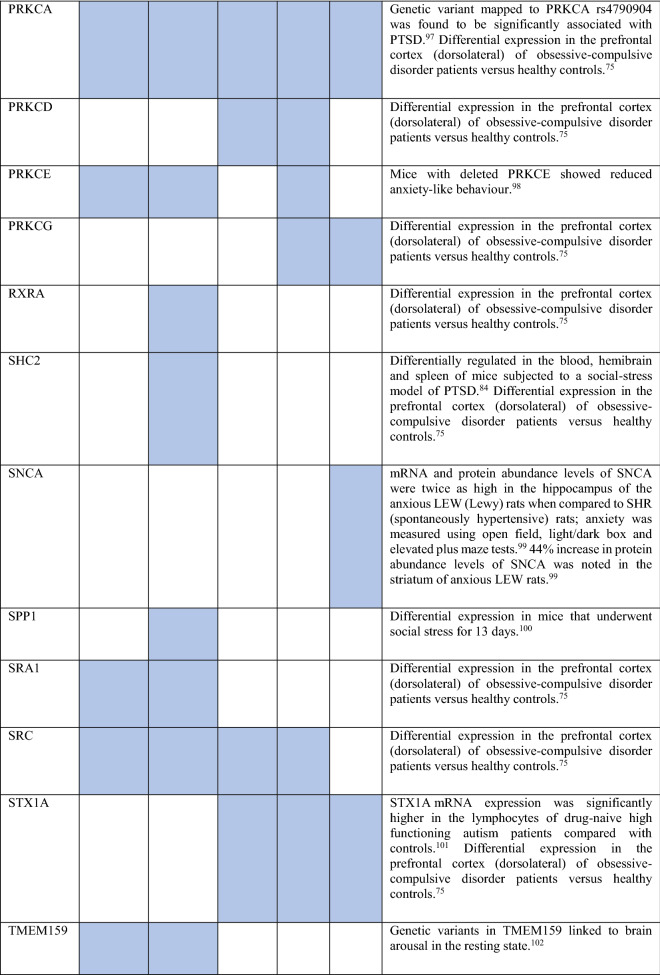
Genes present in AD-SMIs that were expressed in the four key brain regions influential in producing the grouping pattern of ADs (shown in Fig. 4a,c). A colored cell indicates the presence of the particular gene in the corresponding AD-SMI. The final column presents the literature evidence supporting the potential role of the given genes in anxiety etiology.

## Discussion

Neuroimaging, neurochemical and gene-based approaches have provided valuable insights on neurobiological and genetic themes underlying AD etiology^9,103–105^. Regional neural activity has been correlated with anxiety traits such as pessimistic valuation and negative affectivity associated with facial expression^16,17^. Since resting-state functional connectivity in the brain shares substantial overlap with correlated regional gene expression patterns^18–23^, perturbed region-specific transcriptional signatures could underlie cognitive and emotional states in anxiety^24^. In order to capture the complexity of neuropsychiatric phenotypes, such transcriptional signatures need to be examined within an interactomic framework, in which the effect of a perturbed gene spreads in the protein interactome and affects other proteins and the biological processes mediated by them. We thus reasoned that perturbations in region-specific transcriptional profiles may be reflected in the interactome and could underlie region-specific activity in ADs. Several studies have identified higher-order biological relationships existing among genes from the functional landscape of the interactome^30–33^. In this study, we adopted a data-driven interactome and transcriptome-based approach to elucidate common and distinctive neurobiological themes underlying six types of ADs.

Clustering of ADIs based on the region-specific expression of their constituent genes failed to reveal any clustering among OCD, GAD, SAD and panic disorder. Moreover, analysis of component loadings correlated with PC1 and PC2 revealed that this approach failed to capture regional specificities underlying the ADs (Fig. 2b and Supplementary Fig. S1b). Therefore, contrary to our speculation, the clustering of ADs was not directly evident at the level of their interactomes (Fig. 2c). It was evident only after filtering out genes that showed no overlap with the interactomes of genes specifically expressed in striatal compartments called the striosome and the matrix (Figs. 4c, 6c) and retaining only those genes that did show an overlap (Fig. 3).

In our study, striatum-expressed genes, which are closely connected to modulators of striatal development, were found to co-occur in all the ADIs (Supplementary Fig. S2). Genes active in the serotonergic synapse (KEGG^72^) were enriched (*p* value = 1.75E−07) among these 36 genes, namely, APP, GNAI2, GNAI3, GNB5, KCNJ9, MAPK1, MAPK3, PRKCA and SLC6A4 (serotonin transporter). The fact that selective serotonin reuptake inhibitors (SSRIs) function as broad-spectrum drugs across ADs (and the highly co-morbid major depressive disorder)^106^, led us to prioritize striatum as a focal region, with potential involvement in neurodevelopmental underpinnings of AD^1^. Two factors seemed to support this suspicion: striatum is (a) anatomically and functionally connected to brain regions that have been extensively studied in the context of anxiety, such as the amygdala, hippocampus and the prefrontal cortex^107^, and (b) involved in key cognitive processes impaired in ADs, such as attention, motivation, fear conditioning and reward prediction error^107^. The main finding in this direction was that each of the ADIs, except the PTSD interactome, shared a preferential and statistically significant overlap with the striosome/matrix interactomes (i.e., SMIs) (Fig. 3).

We then attempted to cluster the ADs based on the region-specific expression of genes overlapping between the ADIs and the SMIs (i.e., AD-SMIs). This revealed clearer clustering patterns among the disorders (Fig. 4c and Fig. 6c), which seemed to be influenced by regional specificities, with some brain regions showing a strong correlation with PC1 and PC2 (Fig. 4b and Fig. 6b). The enrichment patterns of AD-SMIs in specific brain regions may reflect the related brain circuitry, which produces heightened responses to triggers and sequentially generates anxiety (see Supplementary Discussion)^108^. The same set of genes contributed towards the enrichment of each of the AD-SMIs across the four regions that were highly influential in AD-SMI clustering, namely, amygdala, hippocampus, ACC and nucleus accumbens. This may reflect the concerted roles (a) of these regions in the limbic circuit or (b) of neuronal synaptic signaling pathways across these regions (Fig. 7). Another reason could be the inclusion of any gene with high/moderate rather than tissue-specific expression in the expression profile of each region (see Supplementary Discussion).

Our results point to a scenario wherein the functionally distinct striatal pathways constituted by the striosome and the matrix, act as ‘diverging points’ for the etiological differentiation of various ADs. Genetic perturbations associated with a specific AD may (a) modulate the interactome in one of the two compartments or even their progenitor populations, such as LGE (Fig. 6c), (b) influence their functional connectivity with other regions and (c) govern the ‘route’ of development of key anxiety traits. GAD- and OCD-associated traits generated in this manner may include internally generated ruminations (involving the phenomenon of interoceptive awareness^109^), cognitive rigidity^50^ and pessimistic valuation^44,49^, controlled by ACC, which targets the striosome compartment^44^. SAD-associated traits may include heightened emotional responses to neutral stimuli and excessive emotional contextualization during social information processing, controlled by the amygdala that collates information from the sensorimotor cortices^110^, which in turn targets the matrix compartment. The behavioral traits characterizing striosome- and matrix-associated ADs could also arise from differential responses of these striatal compartments to reinforcement contingencies^111^ and differential involvement in resolving motivational conflicts^112^. Both these paradigms are intricately tied to anxiety etiology and influenced by dopamine circuits. We noted that the dopaminergic signaling pathway may be highly influential (Fig. 7b) in producing the AD-SMI clustering seen in Fig. 4a,c. Therefore, it is possible that the bifurcation in ADs may stem from differential dopamine dynamics, namely, differential electrically evoked dopamine release^113^, dopamine levels^114^, dopaminergic innervation^114^ and modulation of state transitions in the striatal compartments^115^, and the preferential striosomal projection to dopaminergic neurons in substantia nigra pars compacta^116^. GABAergic and glutamatergic signaling were noted to be moderately influential in AD-SMI clustering (Fig. 7b). The role of our three key neuronal synaptic signaling pathways is well-supported in anxiety studies (see Supplementary Discussion). Further investigations are necessary to delineate their roles in the association between ADs and the striatal compartments.

Our speculation on the potential effects of genetic perturbations on AD etiology is valid when they are examined as neurodevelopmental disorders, i.e., under the assumption that ADs develop when genetic risk factors affect the formation of neural circuits that mediate anxiety and subsequently modulate their responsiveness to anxiety-inducing events^117^. However, a converse scenario is also conceivable, wherein anxious behavior and adverse events alter gene expression via epigenetic mechanisms and modulate the neural circuits and susceptibility to ADs (e.g., the genes NR3C1^118^ and FKBP5^119^ undergo stress-induced epigenetic alterations)^120^. Hence, it is important to note that our results do not illustrate any cause-and-effect relationships among genetic perturbations, disrupted neural circuits/signaling pathways and ADs.

The PTSD interactome was excluded from our analysis as it did not show any distinctive overlap with either the striosome or matrix interactomes. However, we noted that its mechanism could be mostly, if not entirely, different from the other ADs. Corroborating previous studies^121^, the PTSD interactome showed the highest enrichment for the hippocampus followed by the striatum, substantia nigra and amygdala, a pattern that was not shown by any other AD (Fig. 2c). This may justify its placement in a separate group (trauma and stress-related disorders) in DSM-5, unlike the case of OCD, which has been placed in a separate group (OCD and related disorders) despite its etiology being very closely related to the other ADs.

Specific phobia clustered independently from GAD, SAD, OCD, panic disorder and PTSD in the analysis with ADIs, which was performed without taking the striatal subdivisions into consideration (Fig. 2c). Lower enrichment of cortical regions, including the frontal cortex and ACC, and higher enrichment for the spinal cord and the cerebellum could differentiate specific phobia from the rest of the ADs. However, ADI clustering with the inclusion of the striosome-matrix division led to its grouping with OCD, characterized by lower enrichment for the amygdala and hippocampus compared with SAD and panic disorder (Fig. 4c). Analysis with a dataset containing a larger number of regions again isolated specific phobia from the rest of the ADs (Fig. 6c). This isolation was characterized by a high enrichment of three transitory fetal structures—LGE, MGE and upper rhombic lip—and the cerebellum and the striatum, compared with the rest of the ADs. Together, these results seem to indicate (a) the etiological distinction of specific phobia from the other ADs, possibly driven by the spinal cord and the cerebellum, and (b) a possible association with GAD and OCD during the early stages of striosome-matrix specification, as indicated by its affiliation to LGE, a focal region in this process.

We used an interactome-driven model to examine the interrelatedness of ADs, using AD-associated genes as starting points. Our study (a) demonstrates the validity of the protein interactome as a data integration platform, (b) provides evidence supporting the role of the striatum in AD etiology and (c) proposes striosome-matrix specification as a key process with the potential to explain the neurodevelopmental origins of ADs. However, our study has several limitations, and our results should be interpreted with caution. Firstly, we used cross-species data (in addition to human data) as starting points for the construction of ADIs and SMIs (after mapping it to orthologous human data). This cross-species approach is necessary to connect research in animal models to human ADs and gather a systems-level view of the multiple biological levels that affect AD etiology (gene, local and global neuronal circuitry and behavior). Nevertheless, it is not advisable to draw direct transcriptomic/proteomic/phenotypic equivalences between humans and animal models unless the biological levels are comprehensively characterized in all the species, and a clear equivalence of factors defining a condition such as ‘anxious behavior’ is demonstrated in both the species^122^. Secondly, the reliability of our ADIs could be limited by the variability in differential AD diagnosis across a large number of studies (compiled by DisGeNET) from which the AD-associated genes were extracted. Challenges to the differential diagnosis of ADs include the high comorbidity of ADs, their comorbidity with other psychiatric disorders such as major depressive disorder, bipolar disorder and schizophrenia, and their occurrence with a range of other conditions such as substance use disorders, asthma, thyroid disease and complex partial seizures^120^. Thirdly, our ADIs are static networks, which do not incorporate data at various levels of granularity (cell, tissue and organism) or spatiotemporal points. Therefore at this stage, the interactome model will not be able to account for the complex and dynamic events that influence AD development, ranging from genetic mutations and PPI perturbations to gene-environment interactions, and the varying developmental trajectories of anxiety symptoms. Lastly, further investigations are necessary to characterize anxiety as an emergent property driven by specific neural circuits or neural mass effects. Our study proposes striatum and its subdivisions as one of the several candidate regions that may be prioritized for anxiety research.

In summary, our study reveals distinctive interactome overlaps shared between different ADs and striatal compartments and a bifurcation among ADs that are influenced by key anxiety-associated regions and neuronal signaling pathways. Our study proposes striatum as one of the focal regions for future AD research.

## Methods

### Compilation of genes associated with ADs

Genes associated with six types of ADs (from ref^55^ Fig. 1. Suggested scheme for exploring a suspected anxiety disorder), namely, post-traumatic stress disorder (PTSD), obsessive–compulsive disorder (OCD), generalized anxiety disorder (GAD), social anxiety disorder (SAD), specific phobia and panic disorder were extracted from DisGeNET^56^. A gene-disease association score ≥ 0.01 was chosen to ensure that at least one publication has linked the gene in question with the disease. Note that ‘association’ of a gene with an AD here does not imply ‘causality’ in most cases, and may only indicate an association with disease susceptibility or behavioral endophenotypes of diseases. We compiled a list of 33 genes for GAD, 109 genes for PTSD including chronic PTSD, 134 genes for OCD including obsessions, OCD behaviour, OCD trait and OCD personality, 22 genes for SAD, 22 genes for specific phobia including claustrophobia and tocophobia, and 93 genes for panic disorder including panic disorder with agoraphobia (Supplementary Table S1).

### Construction of interactomes

Protein–protein interactions (PPIs) in the human interactome were compiled from Human Protein Reference Database (HPRD)^59^ and the Biological General Repository for Interaction Datasets (BioGRID)^60^ using the Cytoscape plugin, Bisogenet^123^. The network building options were: organism—Homo sapiens, biorelation type—protein–protein interaction, data sources—BioGRID and HPRD, method—input nodes and its neighbors upto a distance of 1. The extracted interactomes included direct interactors of genes associated with the specific AD and intermediate interactors connecting AD-associated genes. This resulted in the GAD interactome with 595 genes and 3517 PPIs, SAD interactome with 266 genes and 887 PPIs, PTSD interactome with 2119 genes and 19,687 PPIs, specific phobia interactome with 634 genes and 4799 PPIs, OCD interactome with 1718 genes and 15,359 PPIs and panic disorder interactome with 773 genes and 4261 PPIs (Supplementary Data S1). The interactomes were visualized using Cytoscape^124^.

### Construction of striosome and matrix interactomes

Genes differentially expressed in striosome and matrix compartments of the striatum were compiled from Table 1 of Graybiel et al.^47^ In cases where the genes were from other species, the corresponding human genes were retrieved from the Homologene database (https://www.ncbi.nlm.nih.gov/homologene), these genes were mapped to their human homologs (taxon id: 9606). This yielded 49 and 19 striosome and matrix genes, respectively (Supplementary Table S3). The striosome and matrix interactomes with 827 genes and 6274 PPIs, and 213 genes and 779 PPIs respectively were then assembled from the human interactome (Supplementary Data S2), as explained before in the case of ADIs.

### Gene expression enrichment in brain regions

We checked the enrichment of AD-SMI genes among genes expressed in specific brain regions. RNA-Seq data from the brains of adult donors was extracted from GTEx^57^. Genes with high or medium expression (transcripts per million (TPM) ≥ 9) in 13 brain regions were included, provided that they were not housekeeping genes, i.e. genes detected in all the tissues with transcripts per million ≥ 1, as identified in the Human Protein Atlas^58^. TPM is a metric for quantifying gene expression; it directly measures the relative abundance of transcripts. 9638 genes were considered as housekeeping genes. A gene matrix transpose (GMT) file was created with amygdala (1953 genes), ACC-BA24 (2269 genes), caudate nucleus (2229 genes), cerebellar hemisphere (3978 genes), cerebellum (3968 genes), cortex (2706 genes), frontal cortex-BA9 (2872 genes), hippocampus (1949 genes), hypothalamus (2374 genes), nucleus accumbens (2464 genes), putamen (1892 genes), spinal cord-cervical c-1 (2408 genes) and substantia nigra (1949 genes).

For an independent analysis, a GMT file with genes having log(Reads Per Kilobase per Million mapped reads) > 2 in 26 brain regions, that are not housekeeping genes, were compiled from Allen Brain Atlas^69^. This included amygdaloid complex (4278 genes), anterior (rostral) cingulate (medial prefrontal) cortex (7022 genes), caudal ganglionic eminence (4303 genes), cerebellar cortex (4580 genes), cerebellum (4563 genes), dorsal thalamus (4271 genes), dorsolateral prefrontal cortex (4570 genes), hippocampus (hippocampal formation) (4713 genes), inferolateral temporal cortex -area TEv, area 20 (7436 genes), lateral ganglionic eminence (4448 genes), medial ganglionic eminence (4395 genes), mediodorsal nucleus of thalamus (4503 genes), occipital neocortex (4457 genes), orbital frontal cortex (4617 genes), parietal neocortex (4443 genes), posterior (caudal) superior temporal cortex-area 22c (4558 genes), posteroventral (inferior) parietal cortex (4475 genes), primary auditory cortex (core) (4540 genes), primary motor cortex-area M1, area 4 (4545 genes), primary motor-sensory cortex (4488 genes), primary somatosensory cortex-area S1, areas 3,1,2 (4520 genes), primary visual cortex-striate cortex, area V1/17 (4528 genes), striatum (4628 genes), temporal neocortex (4384 genes), upper (rostral) rhombic lip (4480 genes) and ventrolateral prefrontal cortex (4575 genes).

The GMT files served as inputs for a gene over-representation analysis (GSEA) based on the hypergeometric distribution. In this method, the *p* value is computed from the probability of k successes in n draws (without replacement) from a finite population of size N containing exactly k objects with an interesting feature.

$$ {\text{P }}\left( {{\text{X}} = {\text{k}}} \right) = \frac{{\left( {\begin{array}{*{20}c} {\text{K}} \\ {\text{k}} \\ \end{array} } \right)\left( {\begin{array}{*{20}c} {{\text{N}} - {\text{k}}} \\ {{\text{n}} - {\text{k}}} \\ \end{array} } \right)}}{{\left( {\begin{array}{*{20}c} {\text{N}} \\ {\text{n}} \\ \end{array} } \right)}} $$

N = Total number of genes expressed in any brain regions. K = number of genes expressed in a particular brain region. n = number of genes co-occurring in an ADI and SMIs. k = number of common genes between K and n (genes co-occurring in an ADI and SMIs, that are also expressed in a particular brain region). Enrichment ratio is computed as the ratio of k/n and K/N.

### Signaling pathway enrichment in brain regions

WebGestalt was used to compute the distribution of genes involved in a specific signaling pathway in the gene sets that were responsible for the enrichment of the AD-SMIs in highly influential brain regions, and compare it with the background distribution of genes belonging to this pathway among all the genes associated with any pathway in the selected database (KEGG^72^)^73^. Statistical significance of the enrichment was computed using Fisher's exact test, and corrected using the Benjamini–Hochberg method for multiple test adjustment.

### Principal component analysis

Principal component analysis (PCA) was used to capture relationships between the ADIs, and between AD-SMIs. Negative log-transformed *p* values indicating the statistical significance of enrichment of various brain regions in ADIs/AD-SMIs were assembled into a data matrix containing brain regions as rows and ADs as columns; each cell in the matrix contained a −log_10_P value. PCA was performed with a web-based tool called ClustVis (https://biit.cs.ut.ee/clustvis/)^125^. The data matrix was pre-processed to include only those rows and columns that contained less than 70% missing values. The –log_10_P values in the matrix were further centered using the unit variance scaling method, in which the values are divided by standard deviation so that each row or column has a variance of one; this ensures that they assume equal importance while finding the components. The method called singular value decomposition (SVD) with imputation was used to extract principal components. In this method, missing values are predicted and iteratively filled using neighbouring values during SVD computation, until the estimates of missing values converge. The number of principal components computed was equal to the number of column dimensions in the data matrix, i.e. the number of ADIs or gene sets shared between ADIs and SMIs, in our case. PCA essentially transformed our original variables (–log_10_*P*) into uncorrelated variables called principal components. These principal components were ranked in the descending order of the percentage of total variance explained by them. We extracted the first two components, i.e. PC1 and PC2, and plotted the component scores of each tissue on a 2D plane to capture the angle of most variability and delineate grouping patterns based on approximated distances between the scores. Scores corresponding to PC1 and PC2 were plotted along the X and Y axes respectively.

After an initial assessment of potential clusters, we extracted factor/component loadings corresponding to all the brain regions that contributed to the selected principal components. Component loadings are correlation coefficients between the variables in rows and the factors (i.e. PC1, PC2 etc.). The squared value of a component loading gives the percentage of the variance explained by a particular original variable, and essentially its contribution to the principal components. We plotted the loading of each brain region corresponding to PC1 (X-axis) and PC2 (Y-axis) to examine their influence on the grouping patterns observed on the PC plot.

### Clustering analysis

The data matrix of brain regions (rows) and ADIs or AD-SMIs (columns) was subjected to hierarchical clustering using the tool called Heatmapper (http://www.heatmapper.ca/)^126^, in order to check whether the grouping patterns observed in the PC plot are valid. Pairwise distances in the data matrix were calculated using Pearson correlation and they were ‘linked’ using the average linkage method. Dendrograms were generated by merging tissue samples with the smallest distance first, and those with larger distances later. In the average linkage method, the average distance of all possible pairs is considered while clustering^127^.

## Supplementary Information

Supplementary Information 1.

Supplementary Information 2.

Supplementary Information 3.

## Data Availability

ADIs and SMIs analyzed in this study have been made available as Supplementary Data S1 and Supplementary Data S2, respectively.
