# Supplementary material for: Generalized and social anxiety disorder interactomes show distinctive overlaps with striosome and matrix interactomes
Source: Sci Rep. 2021 Sep 15;11:18392. doi: 10.1038/s41598-021-97418-w (PMC8443595; doi:10.1038/s41598-021-97418-w)
Supplement: Supplementary file 1 — Supplementary Information 1. [file 41598_2021_97418_MOESM1_ESM.docx]

## Supplementary Information

Generalized and social anxiety disorder interactomes show distinctive overlaps with striosome and matrix interactomes

Kalyani B. Karunakaran^1^, Satoko Amemori^2^, N. Balakrishnan^1^, Madhavi K. Ganapathiraju^3,4*^ and Ken-ichi Amemori^2*^

^1^Supercomputer Education and Research Centre, Indian Institute of Science, Bangalore, India

^2^Institute for the Advanced Study of Human Biology, Kyoto University, Kyoto, Japan

^3^Department of Biomedical Informatics, School of Medicine, and ^4^Intelligent Systems Program, School of Computing and Information, University of Pittsburgh, Pittsburgh, USA

*Correspondence to: Ken-ichi Amemori ([amemori.kenichi.7s@kyoto-u.ac.jp](mailto:amemori.kenichi.7s@kyoto-u.ac.jp)), Madhavi K. Ganapathiraju ([madhavi@pitt.edu](mailto:madhavi@pitt.edu))

This file contains:

- Supplementary Methods
- Supplementary Notes S1 and S2
- Supplementary Discussion
- Supplementary Figures S1 to S4
- Supplementary Tables S1 to S3
- Supplementary References 1 to 17

**Supplementary Methods**

## Construction of thalamic nuclei interactomes

Genes differentially expressed in five different thalamic nuclei of rhesus monkeys were compiled from Murray et al.^1^ Genes with FC > 1.2 at p-value < 0.05 were considered to be differentially expressed, where FC is fold change of gene expression. On filtering with these criteria, 42, 95, 137, 29 and 181 genes were found to be differentially expressed in anterior dorsal, center median, medial dorsal, pulvinar and ventral dorsal thalamic nuclei respectively. Interactomes of these genes were then assembled as explained before. The anterior dorsal thalamic nucleus interactome consisted of 295 genes and 1532 PPIs. Center median nucleus interactome had 1430 genes and 12813 PPIs. Medial dorsal nucleus interactome had 1243 genes and 10959 PPIs. Pulvinar interactome had 328 genes and 1425 PPIs. Ventral dorsal nucleus interactome had 1579 genes and 14806 PPIs.

**Supplementary Note S1**

**Enrichment of behavioral and motor stereotypes among genes shared between OCD and striosome interactomes**

Genes that overlapped between OCD and striosome showed high enrichment for genes associated with motor and behavioral stereotypes. Specifically, we noted enrichment of genes associated with the Human Phenotype Ontology^2^ term ‘stereotypy’ among genes found in the striosome interactome that overlapped with genes in the OCD interactome (p-value = 0.033, odds ratio = 1.32). Human stereotypical behaviors such as repetitive, compulsive behavior, stereotypical hand wringing, recurrent hand flapping, punding, eye-poking, stereotypical body rocking and tongue thrusting which manifest in a wide range of disorders such as autism, Asperger syndrome, dystonia, Rett syndrome and epilepsy were considered for this analysis. The repetitive pattern of negative decision-making has been linked to striatal stimulation in macaque monkeys^3^, and excessive activation in the striosomes compared with activation in the matrix has been shown to predict the degree of drug-induced motor stereotypy in rats.^4^ The perturbation of closely interconnected OCD and striosomal genes should be experimentally investigated for its potential role in stereotypical behavior manifested in anxiety disorders (ADs), as well as in behavioral and motor stereotypes observed in autism and dystonia.

**Supplementary Note S2**

**Overlaps between thalamic nuclei and anxiety disorder interactomes**

In addition to striatum-expressed genes, thalamus-expressed genes were also enriched in the network of striatal developmental regulators and anxiety-associated genes (**Supplementary Fig. S2**). Therefore, we checked whether genes with specific expression in different thalamic nuclei showed preferential enrichment in anxiety disorder interactomes (ADIs), similar to the observed preferential enrichment of striatal sub-compartments. We compiled a list of genes differentially expressed in the anterior dorsal, center median, medial dorsal, pulvinar and ventral dorsal thalamic nuclei.^1^ The interactomes of these genes were assembled and their overlaps with ADIs were computed. The interactomes of all the five thalamic nuclei showed overlaps with all the ADIs at p-value < 0.05, except in the case of phobia interactome, which did not show overlap with the ventral and dorsal thalamus. This might indicate that, unlike striatal sub-compartments, molecularly distinct subdivisions of the thalamus do not show preferential association with any AD. While this does not rule out the possibility of preferential involvement of sub-compartments of other brain regions in ADs, out of the two brain regions that were overrepresented among all the ADs, striatum, and not thalamus, seemed to be a potential candidate to dissect the etiological divergence of the different sets ofADs.

**Supplementary Discussion**

**Functional connections of striatal compartments with anxiety-associated brain regions**

Striosomes predominantly receive inputs from cortical areas implicated in limbic and evaluative processes such as the posterior orbital, anterior cingulate, and caudal prefrontal cortices.^5^ The matrix predominantly receives inputs from the sensorimotor cortices, the association cortex and the thalamus. Our results raise the hypothesis that distinctive functional congruence of the brain regions directly innervating these striatal compartments, or innervated by regions targeting these compartments, with key aspects of GAD and SAD may explain why they were differentiated in this study. Several observations support this notion.

Striatum receives information from the amygdala and several cortical regions upon exposure to stress stimuli.^6^ In disorders such as GAD, the ‘threat’ may be generated as an ‘internal trigger,’ potentially activating the striosomal compartment directly. Anterior cingulate cortex (ACC) has been implicated in interoceptive attention,^7^ which we suspect is the basis for the ‘internal trigger’ in GAD and OCD. Interoception may be defined as an ‘internal sensation’ of the physiological condition of the body, which may underlie the subjective experience of emotions. It is believed to be critical for maintaining a homeostatic internal state and often viewed as a connector of physiological changes and the affective and cognitive processes in the brain. Pessimistic evaluation is a critical feature in GAD. Several studies suggest that the striosome-related circuit could be related to the causal source of the pessimistic valuation in decision-making. Striatal stimulation and beta oscillation have also been linked to behavioral inflexibility — a key feature in OCD — which manifested as a repetitive pattern of pessimistic valuation.^3^ ACC has been shown to target striosomes and increase decision-making based on pessimistic valuation in macaque monkeys.^8,9^ In rodents, optogenetics inhibition of the prelimbic-striosome pathway enhanced behavioral choices toward an emotionally conflicting outcome.^10^ Further, stimulation of the striatum has also been shown to produce pessimistic valuation.^3^ Discrete interactome overlap of GAD with striosome and high enrichment of genes contributing to this overlap in the ACC may allude to some process in which the ACC is activated by an ‘internal trigger’ (and not the amygdala), on being activated by an ‘internal trigger’ (as opposed to an ‘external trigger’), activates the striosomal compartment of the striatum and promotes pessimistic valuation.

On the other hand, threat in the case of SAD may be generated as an ‘external trigger’ that may activate the amygdala, which may then activate the striatum. The key point to be noted here is that the amygdala collates information from sensorimotor cortices,^11^ which also preferentially target the matrix compartment. Discrete interactome overlap of SAD with matrix and high enrichment of the overlapping genes in the amygdala may reflect parallel sensorimotor innervation of the amygdala and striatal matrix. Moreover, the amygdala plays a role in emotional contextualization of sensory stimuli during social information processing, by collating information from sensorimotor regions and the prefrontal cortex.^11^ Amygdala activation has been associated with SAD. Individuals with SAD show increased amygdala activation while viewing neutral facial expressions, as well as when neutral faces are paired with aversive stimuli compared with healthy controls.^12,13^

**Conservative gene sets responsible for AD-SMI enrichment in influential brain regions**

Four spatially separated regions seemed to be highly influential in the grouping pattern of AD-SMIs, namely, amygdala, hippocampus, nucleus accumbens and ACC (**Fig. 4b**). Interestingly, the same set of genes seemed to be responsible for the enrichment of each of the AD-SMIs across these regions (**Fig. 8**). This observation can be interpreted in multiple ways. Firstly, the expression profiles examined for each of the brain regions only excluded genes showing low expression (transcripts per million < 9) and housekeeping genes; any gene that otherwise showed high/moderate expression in a particular brain region was included, irrespective of whether it exhibited ‘tissue-specificity’ in that region compared to other regions (as defined by BaseSpace Correlation Engine^14^) or not. A gene is considered to be specific to a particular tissue, if the decrease in its expression in each of the other tissues relative to the tissue of interest is greater than 0.8, among a list of tissues ranked by expression intensity. As a result, a scenario wherein several genes are shared among different regions is expected. Nevertheless, it has to be noted that none of the ADIs nor AD-SMIs was enriched for ‘tissue-specific’ genes defined by this criterion. Secondly, the conservative set of genes could reflect concerted functions of the four regions in the limbic circuit, and the perturbation of these genes in each AD-SMI in this circuit. Thirdly, functional enrichment analysis suggests a concerted role for dopaminergic/GABAergic/glutamatergic signaling pathway (**Fig. 7b,c**) in the four brain regions, at least from the perspective of the AD-SMIs. The latter two possibilities cannot be fully ascertained unless the gene-wise correlation of expression levels across different brain regions are investigated.

**Role of three influential synaptic signalling pathways in anxiety etiology**

We noted that the dopaminergic signaling pathway may be highly influential (**Fig. 7b**) in producing the AD-SMI clustering seen in **Fig. 4a** and **Fig. 4c**. Dopaminergic signaling has been linked to anxiety etiology in regions that were identified to influence AD-SMI clustering in our study. Variations in trait anxiety in a non-clinical population have been correlated with variability in dopamine release in the amygdala and ACC.^15^ Decreased dopamine transporter (DAT) density and DRD2/3 binding capacity, and high co-morbidity with Parkinson’s disease (characterized by degeneration of dopaminergic neurons) highlight the role of dopamine in SAD.^16^ Deep-brain stimulation of the dopamine-rich nucleus accumbens ameliorated symptoms in several ADs including OCD.^17^ In our study, GABAergic and glutamatergic signaling were noted to be moderately influential in AD-SMI clustering (**Fig. 7b**). Reduced GABA levels in basal ganglia and ACC (along with occipital cortex) have been observed in panic disorder patients with a family history of ADs and affective disorders.^16^ Symptomatic alleviation of GAD has been observed with GABA receptor agonists that enhance inhibitory GABAergic signaling and glutamate receptor antagonists that inhibit excitatory glutamatergic signaling.^16^ Reduction of anxiety behavior on treatment with drugs limiting glutamate availability has been noted in fear paradigms administered on animal models as well as on human subjects.^16^ Correlation of symptom severity with higher glutamate to creatine ratio in the ACC has been noted in SAD patients.^16^

**
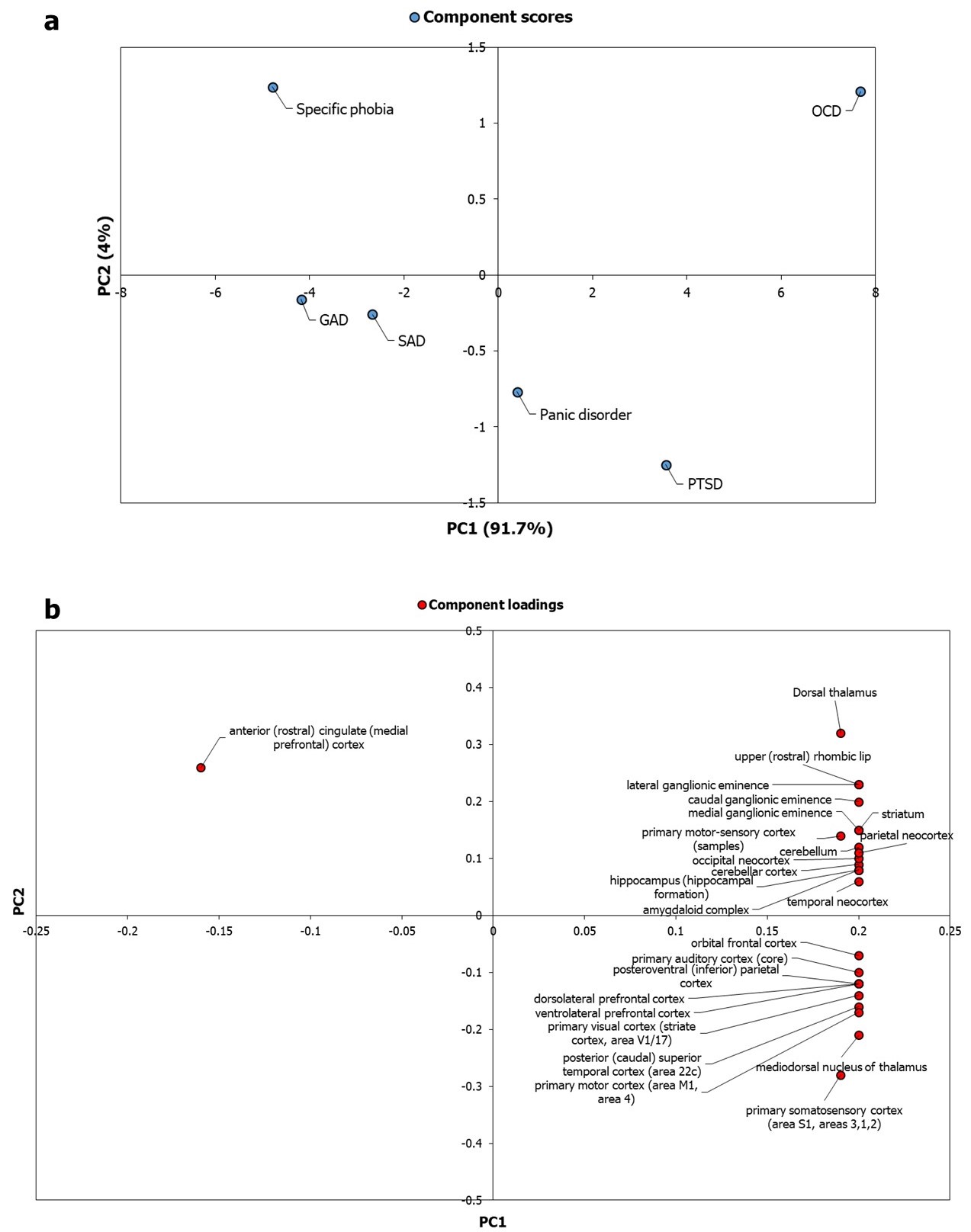
**

**Supplementary Figure S1: PCA of ADIs based on enrichment patterns in the brain (Allen Brain Atlas) failed to capture regional specificity.** (a) Principal component analysis was performed with the p-values of enrichment of the ADI genes in 26 brain regions compiled from GTEx. P*-*values were transformed to –log_10_P values and a matrix with brain regions (rows) and ADs (columns) was constructed out of these log-transformed values. Unit variance scaling was applied across this matrix. Single value decomposition (SVD) with imputation was used to extract the principle components (PC). Component scores (n = 6) corresponding to PC1 and PC2 explaining 91.7% and 4% of the total variance were plotted along X and Y axes respectively. (b) Component loadings of 26 dimensions, i.e. brain region, contributing to PC1 and PC2 shown in (a) were plotted along X and Y axes respectively. Relatively equal and moderate contribution of all the brain regions, except the anterior cingulate cortex, shows that ~96% of the variance captured by PC1 and PC2 plotted in (a) may not have reflected regional specificities of the ADs.


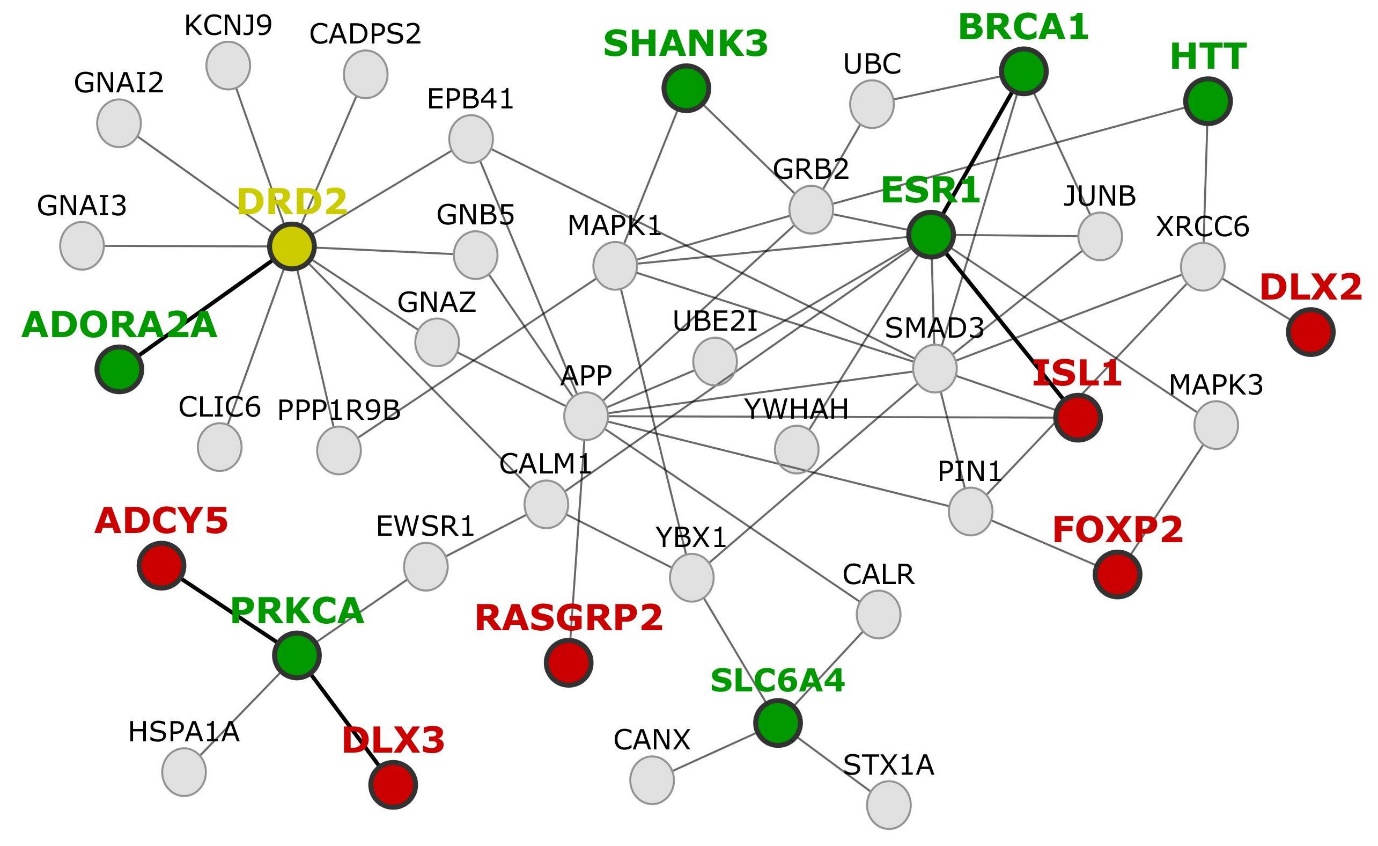


**Supplementary Figure S2: Close interconnections of striatal development-associated genes and AD-associated genes set striatum up as a focal brain region in anxiety etiology.** The diagram shows that proteins encoded by genes involved in striatal development (red-colored nodes) are closely interconnected with genes associated with ADs (green colored nodes) either through intermediate interactors (grey colored nodes) or through direct protein-protein interactions (depicted as thick edges). Genes that are associated with striatal development, as well as ADs, are shown as yellowish green colored nodes. Nodes (circles) depict proteins. Edges (lines) between the nodes depict protein-protein interactions. These interconnections between neurodevelopmental origins of striatum and ADs at the interactome level set striatum up as a potential focal point for investigating the etiology of anxiety. The network diagram was created using Cytoscape (version 3.7.2).

**
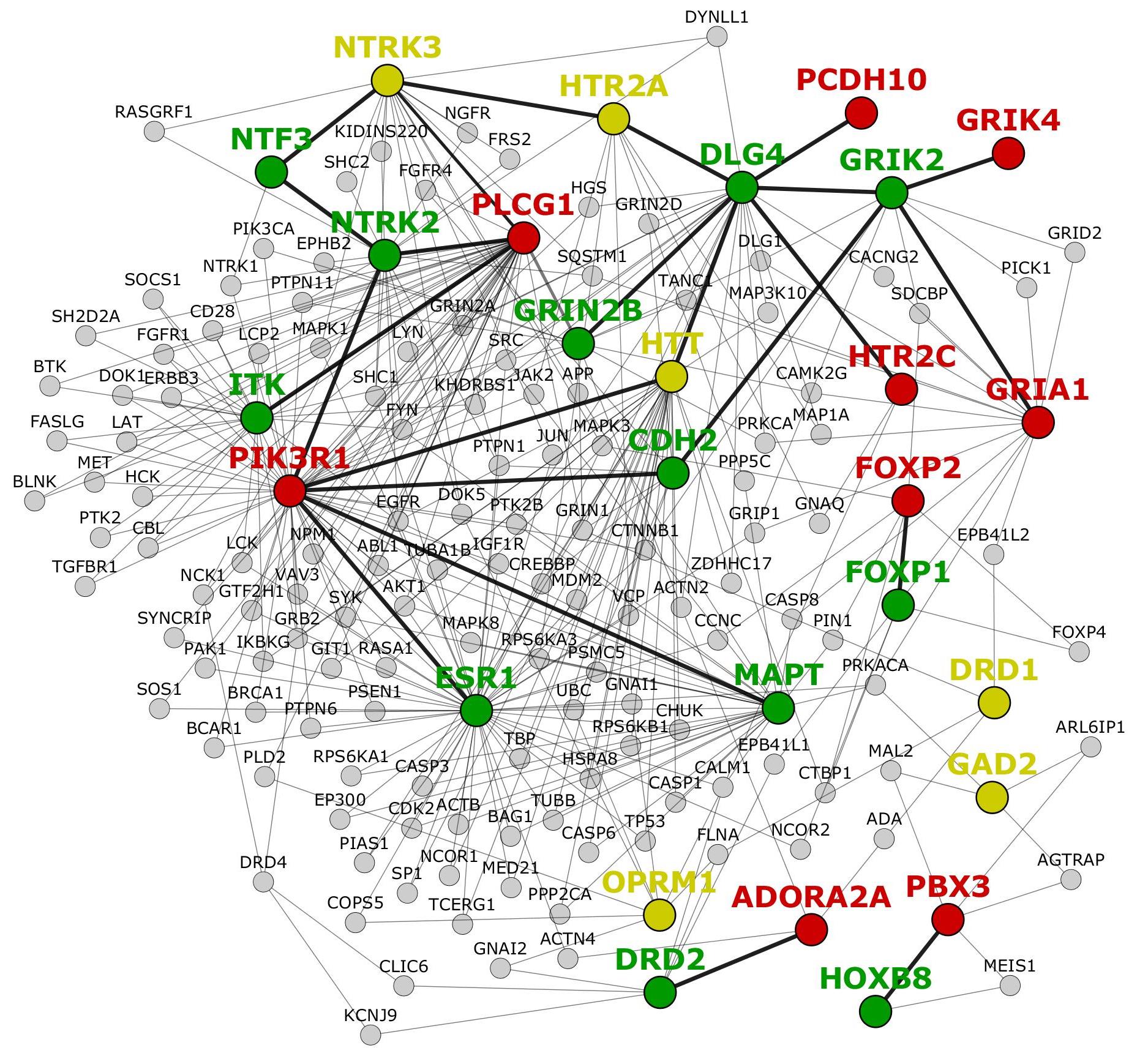
Supplementary Figure S3: OCD-associated genes are closely connected to genes differentially expressed in striosomes.** Nodes (circles) depict nodes and edges (lines) between the nodes depict protein-protein interactions. Genes differentially expressed in the striosome are shown as red-colored nodes, whereas genes associated with OCD are shown as green colored nodes. Genes that are differentially expressed in the striosome as well as associated with OCD genes are shown as yellowish green colored nodes. Thick edges depict direct connections between OCD and striosome genes. The network diagram was created using Cytoscape (version 3.7.2).


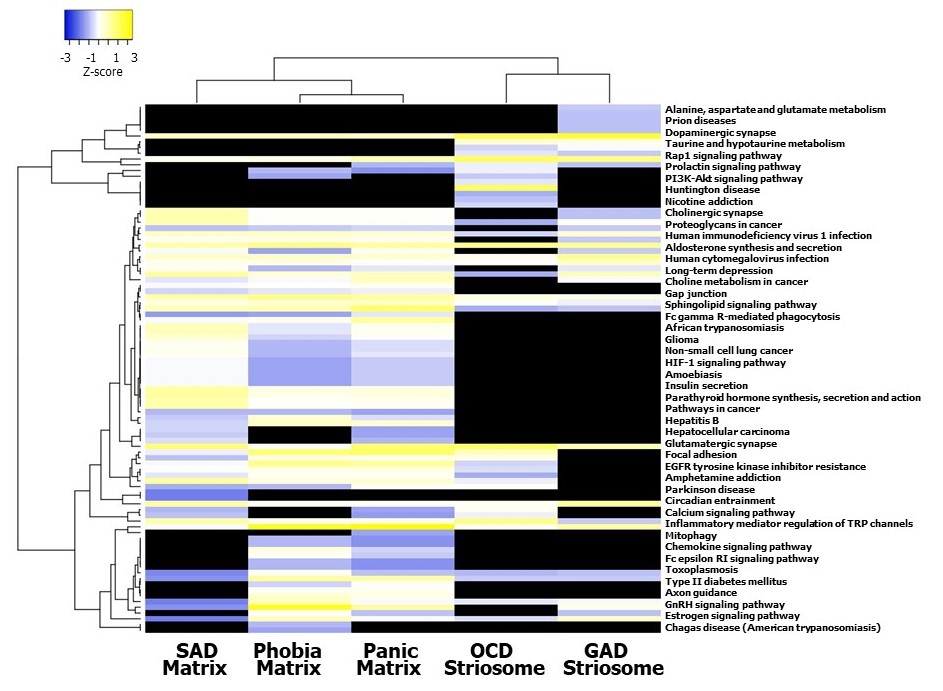
**Supplementary Figure S4: Clustering analysis of gene sets expressed in influential brain regions revealed bifurcation of AD-SMIs.** Gene sets in GAD-striosome, OCD-striosome, phobia-matrix, SAD-matrix and panic-matrix that showed moderate/high expression in the amygdala, hippocampus, ACC and nucleus accumbens were selected, and the KEGG pathways enriched in them were identified. Pathway enrichment of these gene sets are represented in the form of a heatmap. Specifically, normalized Z-scores computed based on the –log_10_ transformed p-values indicating the statistical significance of pathway enrichment of these gene sets are shown in the figure. Z-scores indicate relative enrichment of specific pathways in the gene sets, and are computed based on the number of standard deviations that separate a given p-value from the mean. Clustering was performed using the hierarchical clustering method with average linkage. The dendrograms were derived from the clustering analysis based on computation of Pearson correlation coefficients between the data points. In contrast with **Fig. 4c** and **Fig. 6c**, phobia-matrix clustered alongside SAD-matrix and panic-matrix. The clustered heatmap was created using Heatmapper (<http://www.heatmapper.ca/>).

**Supplementary Table S1: Lists of genes associated with six types of anxiety disorders curated from DisGeNET**

| **Anxiety disorder** | **Genes** |
| --- | --- |
| Obsessive compulsive disorder | ABO, ADNP, AFF2, AQP6, ATF7IP, BAZ1B, BDNF, BTBD9, C9orf72, CACNA1C, CCKBR, CDH17, CDH2, CDH9, CDK20, CHD7, CLIP2, CNTNAP2, COASY, COMT, CRH, CSF2, CYFIP1, CYP2C19, CYP2D6, CYP2E1, DLG4, DLGAP3, DMPK, DNASE1L3, DNM3, DRD1, DRD2, DRD3, DRD4, EHMT1, ELN, ESR1, ESR2, FMR1, FOXP1, FRAXE, GABBR1, GAD2, GJB2, GRIA3, GRIK2, GRIN2B, GRN, GTF2I, GTF2IRD1, GTS, HDAC8, HIVEP1, HNRNPH2, HOXB8, HTR1B, HTR1D, HTR2A, HTR3A, HTR3C, HTR3D, HTR3E, HTT, ITK, KCTD17, KIF22, KMT2A, KRT7, LAMC2, LCT, LIMK1, LINC02395, MAOA, MAPT, MECP2, MED12, MEIS2, MIR485, MLXIPL, MOG, NCSTN, NIPBL, NPS, NPSR1, NTF3, NTRK2, NTRK3, OCRL, OLIG1, OLIG2, OMD, OPRM1, OR2AG1, OXTR, PAH, PANK2, PBX1, PGC, PHOBS, POMC, POU1F1, PPIG, PPM1D, RAD21, RFC2, RYR3, SCLY, SEMA3E, SETD5, SGCE, SHANK3, SLC1A1, SLC22A3, SLC6A3, SLC6A4, SLITRK1, SLITRK5, SMC1A, SMC3, TAL1, TBL2, TDO2, THAS, TNF, TOR1A, TPH2, TRIO, TSPO, TTC19, TUBGCP5, UBE3A, UCP2, XK |
| Post-traumatic stress disorder | ACE, ACTB, ADAMTS2, ADCY8, ADCYAP1, ADCYAP1R1, ADRA2B, ADRB2, ALOX12, ALOX15, ANK3, ANKRD55, APOE, ATP6AP1L, ATP8, BAG3, BAX, BCL2, BDNF, BRCA1, CLOCK, CNR1, COMT, CPT1B, CRHR1, CRHR2, CRP, DBH, DICER1, DNMT1, DRD2, DRD3, DUSP2, EDEM1, EGR1, ENDOU, ERAL1, ESR1, EXOSC6, F8, FAAH, FAM49B, FKBP4, FKBP5, FLT4, FMR1, GAPDH, HSP90AA1, HTR1A, ICAM1, IFNG, IL12B, IL1B, IL2, IL6, INPP1, KLF6, LINC02210-CRHR1, LYZ, MDM2, MFGE8, MIR125A, MIR132, MZB1, ND5, NOS1, NOS1AP, NPS, NPY, NR3C1, NR3C2, NTRK2, OPRL1, OPRM1, OXT, OXTR, P2RX7, PCSK9, PHOBS, PKD2L1, POTEF, PRKCA, PRTFDC1, PSMG1, PTPRVP, RORA, RPS6, S100A10, SCGB1A1, SGK1, SKA2, SLC18A2, SLC6A3, SLC6A4, SRD5A2, STAT5B, STMN1, TBX21, TDO2, THOP1, TLL1, TLR8, TNF, TPH1, TPH2, TSC22D3, VIPR2, WFS1, WWC1 |
| Panic disorder | ACE, ADORA2A, ADRA2A, AKR1C1, AKR1C3, ALAD, ANO2, APOE, ARSI, ASIC1, ASIC2, AVPR1B, BDNF, CALCOCO1, CCK, CCKAR, CCKBR, COMT, CREM, CRH, CRHR1, CRHR2, DAOA, DAOA-AS1, DEAF1, DRD4, DYNLL2, ECT, ELK3, ELN, EPHB1, GABRA5, GABRA6, GABRB3, GAD1, GAD2, GAL, GLO1, GLRB, GPM6A, GRP, GRPR, HLA-B, HLA-DRB1, HP, HTR1A, HTR2A, HTR3A, IKBKE, IL10, INS, LDHA, LDHB, LINC02210-CRHR1, LSAMP, MANEA, MAOA, MBL2, MECP2, MIR148A, MIR22, MIR339, MIR488, MIR491, NPL, NPPA, NPS, NPSR1, NPY, NPY5R, NTRK3, PDE4B, PEPD, PGR, PKP1, PRDM11, PSAP, PTK7, RGS2, RGS7, SDK2, SGCE, SGCZ, SLC6A2, SLC6A4, SNRNP70, SOSTDC1, SRD5A1, TACR1, TMEM132D, TPH1, TPH2, TSPO |
| Generalized anxiety disorder | ALAD, BCL2, BDNF, CASP12, COMT, CRHR1, DRD2, DRD3, DYNLL2, ERN1, GAD1, GAD2, GCK, GJA1, HLA-DQB1, HSPA5, HTR2A, INS, LINC02210-CRHR1, MAOA, NDUFS4, NPY, NR3C1, OSR1, PAWR, PDE1A, PSMD9, PTPN22, RGS2, SLC30A10, SLC30A8, SLC6A4, TPO |
| Social anxiety disorder | ADRB1, ALAD, ARNTL2, BDNF, BEST1, CNTNAP2, CRHR1, CYP2D6, DRD2, DYNLL2, FMR1, HTR1A, MANEA, OXT, OXTR, PSAP, RGS2, SLC2A9, SLC6A2, SLC6A3, SLC6A4, TSC2 |
| Specific phobia | ACE, ADORA2A, CAMKK2, CAT, CREBBP, DRD2, EP300, FMR1, GPM6A, HTR1A, MAOA, MIR22, MIR491, NGF, NPY, P2RX7, PHOBS, PRL, SGCE, SLC6A2, SLC6A4, TPH2 |

**Supplementary Table S2: Genes that co-occur in the interactomes of 6 anxiety disorders.**

| **Gene symbol** | **Gene name** |
| --- | --- |
| ADORA2A | adenosine A2a receptor |
| APP | amyloid beta precursor protein |
| BRCA1 | BRCA1 DNA repair associated |
| CADPS2 | calcium dependent secretion activator 2 |
| CALM1 | calmodulin 1 |
| CALR | calreticulin |
| CANX | calnexin |
| CLIC6 | chloride intracellular channel 6 |
| COMT | catechol-O-methyltransferase |
| DRD2 | dopamine receptor D2 |
| EPB41 | erythrocyte membrane protein band 4.1 |
| ESR1 | estrogen receptor 1 |
| EWSR1 | EWS RNA binding protein 1 |
| GNAI2 | G protein subunit alpha i2 |
| GNAI3 | G protein subunit alpha i3 |
| GNAZ | G protein subunit alpha z |
| GNB5 | G protein subunit beta 5 |
| GRB2 | growth factor receptor bound protein 2 |
| HSPA1A | heat shock protein family A (Hsp70) member 1A |
| JUNB | JunB proto-oncogene, AP-1 transcription factor subunit |
| KCNJ9 | potassium inwardly rectifying channel subfamily J member 9 |
| KMT2A | lysine methyltransferase 2A |
| MAPK1 | mitogen-activated protein kinase 1 |
| MAPK3 | mitogen-activated protein kinase 3 |
| PIN1 | peptidylprolyl cis/trans isomerase, NIMA-interacting 1 |
| PPP1R9B | protein phosphatase 1 regulatory subunit 9B |
| PRKCA | protein kinase C alpha |
| SLC6A4 | solute carrier family 6 member 4 |
| SMAD3 | SMAD family member 3 |
| SORT1 | sortilin 1 |
| STX1A | syntaxin 1A |
| UBC | ubiquitin C |
| UBE2I | ubiquitin conjugating enzyme E2 I |
| XRCC6 | X-ray repair cross complementing 6 |
| YBX1 | Y-box binding protein 1 |
| YWHAH | tyrosine 3-monooxygenase/tryptophan 5-monooxygenase activation protein eta |

**Supplementary Table S3: Lists of genes differentially expressed in the striosome and matrix compartments**

| **Striatal compartment** | **Differentially expressed genes** |
| --- | --- |
| Striosome | CXCR4, DRD1, DRD4, EGR1, KCNIP2, NNAT, NTRK3, PDYN, PHACTR1, HTR2A, HTR2C, DDC, ADORA2A, BCL2, MARCH1, RASGRP1, CALB2, PPP1R1B, NT5E, EPHA7, FAM131B, FOXP2, GNAL, GAD2, GDNF, GRIA1, GRIK4, HTT, ITPR3, KCNIP1, LSAMP, MAP2, OPRM1, NPY2R, TAF1, NR4A1, PBX3, PCDH10, PIK3R1, PLCG1, PLCB4, RELN, SCG2, SRM, CHRM1, CHRM2, CHRM3, CHRM4, CHRM5 |
| Matrix | HTR2A, ACHE, S100G, CALB1, RASGRP1, CNR1, CDH8, CDK5, CHAT, EBF1, EPHA4, GRIA1, HTR4, NQO1, PENK, SOD2, SST, TH, SLC17A6 |

**Supplementary References**

1 Murray, K. D., Choudary, P. V. & Jones, E. G. Nucleus-and cell-specific gene expression in monkey thalamus. *Proceedings of the National Academy of Sciences* **104**, 1989-1994 (2007).

2 Robinson, P. N. *et al.* The Human Phenotype Ontology: a tool for annotating and analyzing human hereditary disease. *The American Journal of Human Genetics* **83**, 610-615 (2008).

3 Amemori, K.-i., Amemori, S., Gibson, D. J. & Graybiel, A. M. Striatal microstimulation induces persistent and repetitive negative decision-making predicted by striatal beta-band oscillation. *Neuron* **99**, 829-841. e826 (2018).

4 Canales, J. J. & Graybiel, A. M. A measure of striatal function predicts motor stereotypy. *Nature neuroscience* **3**, 377-383 (2000).

5 Crittenden, J. R. & Graybiel, A. M. Basal Ganglia disorders associated with imbalances in the striatal striosome and matrix compartments. *Frontiers in neuroanatomy* **5**, 59 (2011).

6 Calhoon, G. G. & Tye, K. M. Resolving the neural circuits of anxiety. *Nature neuroscience* **18**, 1394-1404 (2015).

7 Khalsa, S. S., Rudrauf, D., Feinstein, J. S. & Tranel, D. The pathways of interoceptive awareness. *Nature neuroscience* **12**, 1494-1496 (2009).

8 Amemori, S. *et al.* Microstimulation of primate neocortex targeting striosomes induces negative decision‐making. *European Journal of Neuroscience* **51**, 731-741 (2020).

9 Amemori, K.-i. & Graybiel, A. M. Localized microstimulation of primate pregenual cingulate cortex induces negative decision-making. *Nature neuroscience* **15**, 776 (2012).

10 Friedman, A. *et al.* A corticostriatal path targeting striosomes controls decision-making under conflict. *Cell* **161**, 1320-1333 (2015).

11 LeDoux, J. Rethinking the emotional brain. *Neuron* **73**, 653-676 (2012).

12 Birbaumer, N. *et al.* fMRI reveals amygdala activation to human faces in social phobics. *Neuroreport* **9**, 1223-1226 (1998).

13 Schneider, F. *et al.* Subcortical correlates of differential classical conditioning of aversive emotional reactions in social phobia. *Biological psychiatry* **45**, 863-871 (1999).

14 Kupershmidt, I. *et al.* Ontology-based meta-analysis of global collections of high-throughput public data. *PloS one* **5**, e13066 (2010).

15 Berry, A. S. *et al.* Dopaminergic mechanisms underlying normal variation in trait anxiety. *Journal of Neuroscience* **39**, 2735-2744 (2019).

16 Martin, E. I., Ressler, K. J., Binder, E. & Nemeroff, C. B. The neurobiology of anxiety disorders: brain imaging, genetics, and psychoneuroendocrinology. *Psychiatric Clinics* **32**, 549-575 (2009).

17 Sturm, V. *et al.* The nucleus accumbens: a target for deep brain stimulation in obsessive–compulsive-and anxiety-disorders. *Journal of chemical neuroanatomy* **26**, 293-299 (2003).
